# The interconnectedness of energy consumption with economic growth: A granger causality analysis

**DOI:** 10.1016/j.heliyon.2024.e36709

**Published:** 2024-08-28

**Authors:** Nishitha Perera, Hasara Dissanayake, Diruni Samson, Sajani Abeykoon, Ruwan Jayathilaka, Maneka Jayasinghe, Shanta Yapa

**Affiliations:** a Sri Lanka Institute of Information Technology, SLIIT Business School, New Kandy Road, Malabe, Sri Lanka; b Charles Darwin University, Waterfront Campus, Darwin, Australia

**Keywords:** Renewable energy consumption, Non-renewable energy consumption, Economic growth, Granger causality

## Abstract

In considering today's energy challenges, the link between the usage of renewable and non-renewable energy sources and economic growth has gained substantial policy attention. This research examines the complex relationship between these three variables to understand how non-renewable energy consumption and renewable energy consumption interact and what that means for economic growth. This study uses the Granger causality approach to explore the relationships between non-renewable energy consumption, renewable energy consumption, and economic development. It draws on a comprehensive dataset from the Word Bank database, including 152 nations from 1990 to 2019. The analysis is further disaggregated by four subgroups of countries; least developed, developed, transitional economies and developing countries. The result of this study provides valuable empirical evidence of uni-directional causality running from renewable energy consumption to economic growth and non-renewable energy consumption to economic growth in transitional economies. Furthermore, policymakers should focus on both variables when making decisions because the results show that energy consumption and economic growth are interconnected. Implementing global energy efficiency standards, reducing fossil fuel usage, and adopting regulatory measures are all viable policies for limiting adverse effects on the environment while encouraging economic development.

## 1 Introduction

**Fig. 1:**
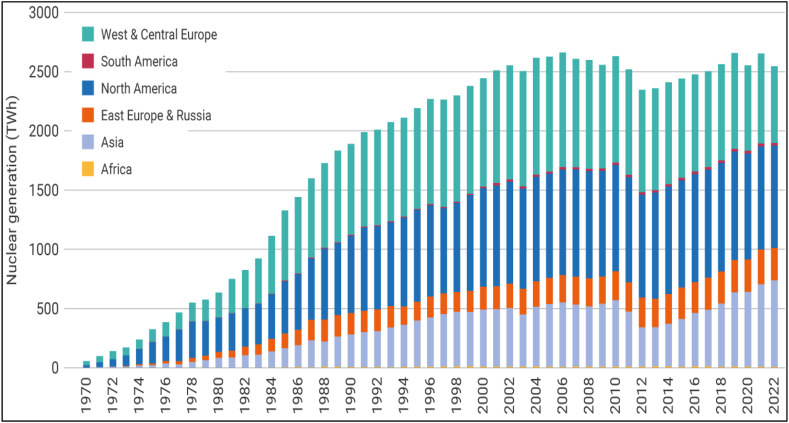
Carbon emissions trend for top nuclear energy regions.

Source: Adopted from World Nuclear Association [1].

Carbon emissions trend for top nuclear energy regions has been shown in Fig. 1. Most of the developed countries are using nuclear power as their primary energy source. According to the statistics, nuclear energy stands as the world's second-largest low-carbon energy source, meeting 10 % of the global energy demand [1].

Economic development depends on the efficient use of energy [2]. However, excessive fossil fuel consumption is hazardous to the environment. More nations are attempting to increase their usage of renewable energy, as is believed, barely creates any greenhouse gases [2]. Nevertheless, all nations - developed or emerging - must continue to expand economically. Employing non-renewable energy could increase productivity and promote economic growth, but it is undoubtedly a significant source of carbon emissions and environmental damage [2]. The use of non-renewable energy sources may force nations to choose between competing interests, reducing pollution or promoting economic growth.

Many scholars have investigated the relationship between economic growth, renewable and non-renewable energy use from various angles. While exploring the direction of the causality between these variables, some studies have found either an uni-directional [3] or a bi-directional causal linkage [4] between REC and economic growth. In terms of the direction of the relationship, some researchers have found a positive linear [5] or a negative linear relationship [6] between the variables under consideration. There are also a limited number of studies that have demonstrated that there is no association [7] or causality [8] between REC and economic growth. The existing research findings are mainly based on the Granger causality [6], Dynamic Ordinary Least Squares (DOLS), Fully Modified Ordinary Least Squares [9], and Autoregressive Distributed Lagged (ARDL) [10] methods. However, most of these studies focus on individual countries or at the global level. Only a limited body of literature is available on specific country groups [11,12].

The study's main objective is to identify the causality between renewable energy consumption (REC), non-renewable energy consumption (NREC) and economic growth between 1990 and 2019 in 152 counties. The analysis will be carried out at the disaggregated level by country group; Least-developed countries (LDCs), developed, transitional economies and developing countries. In doing so, the degree of interaction of the association between REC, NREC, and Gross Domestic Product (GDP) has been examined using correlation analysis. Secondly, a Granger causality approach has been used to analyse the causal link between each of the variables of significance. As a result, the study adds to the current literature in three distinct ways; (1) while there is a substantial body of literature on this topic, only a limited body of literature employed the same approach and variables for various nations, allowing for meaningful comparisons. However, current study discusses the causal relationship between REC, NREC and economic growth for 152 countries to address this knowledge gap. (2) in addition to commonly used time-series econometric techniques, the current study utilises several methods, such as impulse-response functions (IRF) plots, violin graphs, and filled maps, which had not been actively used in existing research; and (3) the current study extends the analysis using the Panel Vector Auto-Regression (pVAR) method to determine the stability criteria, which has been rarely used in the existing literature.

The remainder of this paper is organised as follows: A review of earlier empirical research is provided in the next section, followed by a section on data and techniques describing the Granger causality method. The empirical findings and a discussion of the following investigation are presented next. A conclusion incorporating the study's entire perspective is provided at the end.

## 2 Significance of the study

Switching to sustainable and renewable energy sources has become a top priority due to growing worldwide concerns about climate change. Policymakers, businesses, and society must comprehend how renewable and non-renewable energy affect economic growth. This study aims to illuminate a sustainable and prosperous future by investigating the interactions between different energy sources and their consequences for economic development using data from 152 countries from 1990 to 2019. The study categorises the countries under investigation as LDCs, developed, transitional economies and developing countries to examine the relationship between REC, NREC and GDP growth.

Compared to non-renewable energy sources such as fossil fuels, renewable energy sources such as solar, wind, hydro, and geothermal offer several advantages. By utilising renewable energy sources, nations can reduce their reliance on finite resources, carbon emissions, and air and water pollution. The study provides further insights into the environmental advantages of adopting renewable energy sources and highlights its potential to support long-term sustainability objectives.

Policymakers can use the insights from this study to create energy plans that balance economic development with environmental sustainability. This research aims to guide policy decisions, facilitate the identification of best practices, and highlight potential impediments or challenges one may encounter during the clean energy transition by examining nations' experiences that have effectively incorporated renewable energy into their energy mix. To summarise, the knowledge generated in this study enhances informed decision-making. It expedites the transition to a more sustainable and prosperous future by showcasing the potential of renewable energy sources to spur economic growth while reducing climate change.

## 3 Theoretical framework

The relationship between REC, NREC and economic growth is explained by three theoretical frameworks, namely, (1) the resource curse concept, (2) the energy transition theory and (3) institutional theory.

The resource curse concept suggests that nations that are abundant in non-renewable resources, like oil and gas, may face negative economic and social consequences [13,14]. Over-reliance on fossil fuels can lead to the Dutch disease,^1^ which entails an economy dependent on resource exports, hindering diversification and causing instability. It underscores the importance of diversifying the energy mix towards renewables. Additionally, it emphasises the dangers associated with extracting renewable energy resources.

The energy transition theory recognises how energy transitions' technological, economic, political, and social aspects interact [15]. This theory can be used to analyse market forces, public acceptability, the impact of policy frameworks, and factors that promote and hinder the use of renewable energy sources. Researchers can evaluate the potential for renewable energy to support economic growth while reducing reliance on non-renewable sources by comprehending the complex nature of energy transitions. It is a platform to focus on their own REC for a better green future.

The institutional theory emphasises the importance of institutions, laws, and regulations in influencing economic behaviour and results [16]. This theory can be used to explain the impact of institutional frameworks on the adoption of renewable and non-renewable energy sources. Institutional variables that affect the competitiveness of various energy sources and help create a climate for deploying renewable energy include government policies, subsidies, laws, and international agreements. Insights into the policy mechanisms required to enable sustainable economic growth through renewable energy can be gained by looking at the institutional aspects. These findings are presented while establishing the connection between energy use and economic growth using these three well-known theories.

## 4 Literature review

A thorough examination of the literature was undertaken to conduct this study, focusing primarily on renewable and non-renewable energy's impact on economic growth for 152 countries, dwelling on the four-country subgroups: LDCs, developed, transitional economies, and developing countries. According to the United Nations, LDCs are low-income countries that face severe structural obstacles to achieve sustainable development. They are susceptible to environmental and economic shocks and have relatively low levels of human resources. Countries with industrialised economies transitioning to market economies are known as transition economies. Several countries in transition economies, Europe, a few former Soviet republics, and Russia, are among them. A nation with an undeveloped industrial base, a moderate to low Human Development Index, and a relatively poor living level is said to be developing. Conversely, a developed nation often has higher GDP per capita, industrialisation level, average standard of living, and coverage of modern infrastructure. This section will provide a critical review of the literature to emphasise the research findings, point out any drawbacks and knowledge gaps, and highlight the contribution of the research.

### 4.1 Least-developed countries

The relationships between economic growth and energy use in LDCs have only been lightly researched. Through the application of Granger causality analysis and mediation models in Ghana, the authors revealed that REC had a direct impact on economic growth between 1990 and 2015. Moreover, the results of the nonlinear autoregressive distributed lagged model (NARDL) test based in Rwanda revealed that REC positively influences economic expansion [17]. Furthermore, in this study, positive and negative shocks have an uni-directional causal influence moving from agriculture and capital to economic growth. A study examining the determinants of REC in thirty-two African nations using the DOLS econometric approach found that financial independence and well-being are the primary factors influencing the percentage of renewable energy in overall energy consumption [18].

### 4.2 Developed countries

The research on economic growth and the use of renewable energy has been described as uni-directional and bi-directional interactions as well as the positive and negative impacts between the variables. Some studies of European countries has been identified that there is a bi-directional relationship between REC and economic growth by using wavelet analysis, Granger causality and descriptive analysis, cluster analysis and ARDL methods [4,12,19]. Similarly, an uni-directional relationship from GDP growth to REC was found in Italy based on the Granger causality method [20]. Additionally, using the ARDL and the Granger causality tests in 2014, it was discovered that Italy has an uni-directional causality from REC to economic growth in the long run [3]. Granger causality analysis for 39 high-income European and Central Asian countries indicate uni-directional Granger causation from GDP to REC [21]. Using Granger causality analysis, some researchers have identified the uni-directional causal relationship running from GDP e to REC, proving that ongoing economic growth is accompanied by a steady rise in energy consumption in Italy [22]. The same argument has been established for Germany, where it has shown that there is a significant long-term causal impact on economic growth by REC [23]. The effect of REC on the price of fossil fuels has been investigated [24]. The research showed evidence of both short- and long-term causation between the price of coal and natural gas and the use of renewable energy. This supports the alternative sources between renewable and non-renewable power sources. However, there is no Granger causal link between the price of crude oil and the use of renewable energy sources. Furthermore, a study on Scandinavian countries using a panel analysed methodology has analysed the relationship between REC and economic growth [25].

Investigating whether there are positive or negative relationships between the variables is another area of research. Some studies based on the European Union and Organisation for Economic Co-operation and Development (OECD) have found that REC harms the GDP growth rate [26,27]. The analysis done by a researcher, using ARDL approach reveals that REC in the long run leads to enhanced sustainable development and a decrease in environmental impacts [28]. However, 38 countries based on Renewable Energy Country Attractiveness Index have proved that NREC, REC has a positive effect on economic growth through the research done by the data collected from the World Bank [9]. The same has been confirmed by the research done for European countries, by the data collected from the World Bank [29]. Furthermore, this study also demonstrated that REC positively impacts GDP growth. According to a past research, electricity, fossil fuel use, and economic growth all have an effect on environmental damage in six Asian developed nations, with economic expansion having a positive influence on REC [30]. The authors revealed that the combination of economic complexity and natural resources adds to environmental quality in nations with high institutional quality levels between 1995 and 2017 [31]. Similarly, in countries with high GDP, renewable energy tends to comprise a larger share of final consumption [32]. The authors concluded that the ratification of members of the North American Free Trade Agreement is demonstrated to boost economic growth but restrict environmental sustainability for its members in the long run between 1990 and 2018 [33]. The study highlights the possibility for developed nations to increase their REC consumption.

### 4.3 Transitional economies

There are minimal literature reviews about transitional economies. Only two have been identified by the authors. A study has revealed no significant relationship between economic growth and renewable energy in Russia using the Vector Error Correction Model Granger causality for the years spanning from 1990 to 2014. The same study has also proved that Russia's economic growth and financial development are causally related [34]. However, some researchers found an uni-directional relationship from GDP to REC and from GDP to NREC in a cross-country study of the least developed countries during 1990–2019 [35]. Few studies have examined the connection between renewable energy and economic growth in transitional economies.

### 4.4 Developing countries

In light of recent studies on the link between REC and economic growth in developing countries, the authors have found that there is a bi-directional causality between REC and economic development in 80 developing countries across the world in the period of 1990–2012 [36]. Similarly, based on the Granger causality analysis, a study found a bi-directional causal relationship between economic growth and the use of renewable energy in China [37]. Researchers identified bi-directional causation between globalisation and renewable energy; green investment and renewable energy; and financial development and renewable energy in MINT nations (Mexico, Indonesia, Nigeria, and Turkey) [38]. In the African Organisation of Petroleum Exporting Countries member countries, a study has identified that there is a bi-directional relationship between NREC and economic growth both in the long and short run, based on the analysis of the Granger causality test [39]. A research project based on 42 developing nations has revealed a long-run uni-directional causal association linking REC to economic growth [40]. The findings of the causality test indicate a one-way causality from NREC to economic growth in E7 countries [41]. Author observed that NREC Granger cause sustainable development is validated by causality analysis in Pakistan [42]. In Bangladesh, researchers have established an uni-directional causality between GDP and energy usage in both the short- and long terms [43].

Based on Granger causality analysis, some studies have found that using renewable energy boosts economic growth in Thailand and Pakistan [5,44]. Similarly, a study based on Brazil, Russia, India, China, and South Africa (BRICS) countries found that NREC positively impacted economic growth from 1992 to 2016 [45]. In 41 European countries and 37 African countries, by using panel data analysis, researchers have identified that both short and long-term economic growth and the use of renewable energy positively correlated with each other [46,47].

Moreover, research based on the following eleven countries and analysis of the non-parametric panel data approach demonstrates that REC positively impacts economic growth [48]. According to the findings of the analysis, the authors conclude that renewable energy is a panacea for sustainable development in the face of India's economic growth direction [49]. On the other hand, in South Africa, from 1971 to 2015, authors have suggested a negative sign between the consumption of renewable energy and economic growth [6]. According to the authors economic expansion, fossil fuel energy use, and agricultural activities have a negative impact on environmental sustainability in South Africa, demonstrating a trade-off between economic growth and environmental quality from 1975 to 2020 [50]. Similarly, it was also found that REC negatively impacted economic growth in Ghana, fifteen West African countries and sixteen Asian economies, correspondently, in the aforementioned studies [[51], [52], [53]]. Additionally, a study based on NARDL has suggested that NREC negatively and significantly impacted economic expansion throughout the years 1970–2018 [10]. However, several studies concluded that no connection between REC and NREC and economic growth [2,7,8,54]. Unlike developed nations, the results showed that renewable energy in developing countries plays a significant role in future economic growth.

In conclusion, despite significant research exploring the connection between REC, NREC and economic expansion, the causality, and the course of the association between the attributes are not universally agreed upon. Empirical data are absent for countries in the aforementioned four country classifications. The new study fills this hole in the body of knowledge by considering 152 countries worldwide.

## 5 Data and methodology

This section goes through the data resources and statistical approaches of the research.

### 5.1 The data

This study also investigates the relationship between REC, NREC, and economic growth in 152 nations from 1990 to 2019. “Appendix A” contains the data file that was utilised for the investigation.

The World Bank database is the source of the information for the empirical study, as shown in Table 1. The body of current literature employed the yearly GDP growth rate to measure economic growth. The term “REC” refers to renewable energy consumption expressed as a percentage of all final energy consumption; the word “NREC” is computed as 100%-REC. It is important to note that we have utilised imputed values for estimates because specific time series had missing data, as explained below. The anticipated numbers are all determined using the nearest five years average. North America (2019, 2018, 2017, and 2016), The United Arab Emirates, Micronesia Federal States, The Marshall Islands (1991 and 1990), and Namibia (1990) have predicted data for REC and NREC. Based on GDP growth from previous years, the GDP of Syria Arab Republic (2019), Kiribati (1995 and 1991), Hungary (1991 and 1990), and Slovak Republic (1992, 1991 and 1990) were estimated in this study. Armenia, Azerbaijan, Belarus, Greece, Kazakhstan, Namibia, North Macedonia, Poland, Romania, Slovak Republic, and Yemen Republic (1990), all missing the GDP growth forecast. Thirty-eight nations were classified as LDCs, 29 as developed, 13 as transitional economies, and 72 as developing countries.

**Table 1:** Data and Origins of the attributes.

Variable	Source	Unit of Measure
REC	World Development Indicator https://data.worldbank.org/indicator/EG.FEC.RNEW.ZS	% of total final energy consumption
NREC	World Development Indicator https://data.worldbank.org/indicator/EG.FEC.RNEW.ZS	100 - % of total final energy consumption
GDP growth	World Development Indicator https://data.worldbank.org/indicator/NY.GDP.MKTP.KD.ZG	GDP annual %

### 5.2 The model

The panel Granger causality [55] for the nation categories has been used to establish the causal relationship between REC, NREC, and economic growth. Therefore, the direction and magnitude of causation will be determined using Equation (1). Furthermore, we ran the Levin-Lin-Chu (LLC) unit root test [56] to see if the data set was stationary. The test of stability [[57], [58], [59]] was performed to determine the stability of REC, NREC and GDP.

(1) $$Y_{i,t}=\sum_{k=1}^{\rho }\beta_{i}Y_{i,t-k}+\sum_{k=0}^{\rho }\theta_{k}X_{i,t-k}+u_{i,t}$$

where time and area are represented by the variables i and t, k is the number of lags, and u_i,t_ is the error term. Y is the dependent variable; X is the independent variable. It is hard to distinguish between indirect and direct variables since the REC, NREC, and economic growth were employed to assess the causal link in this study under both directions. In the investigation, lags in the model in Equations (2), (3), (4), (5) were also eliminated using data differences. The estimated approaches were as follows:

(2) $${\text{DGDP}}_{i,t}=\sum_{k=1}^{\rho }\gamma_{i}{\text{DGDP}}_{i,t-k}+\sum_{k=0}^{\rho }\pi_{k}{\text{DREC}}_{i,t-k}+m_{i,t}$$

(3) $${\text{DREC}}_{i,t}=\sum_{k=1}^{\rho }\beta_{i}{\text{DREC}}_{i,t-k}+\sum_{k=0}^{\rho }\sigma_{k}{\text{DGDP}}_{i,t-k}+v_{i,t}$$

(4) $${\text{DGDP}}_{i,t}=\sum_{k=1}^{\rho }\vartheta_{i}{\text{DGDP}}_{i,t-k}+\sum_{k=0}^{\rho }\omega_{k}{\text{DNREC}}_{i,t-k}+s_{i,t}$$

(5) $${\text{DNREC}}_{i,t}=\sum_{k=1}^{\rho }\lambda_{i}{\text{DNREC}}_{i,t-k}+\sum_{k=0}^{\rho }\theta_{k}{\text{DGDP}}_{i,t-k}+u_{i,t}$$

where DREC, DNREC and DGDP are the initial differences of REC, NREC and GDP, respectively. $\gamma_{i},\beta_{i},\vartheta_{i}$ and $\lambda_{i}$ are regression coefficients, while $\pi_{k},\sigma_{k},\omega_{k},{\text{and}\;\theta }_{k}$ are constants for *k* ∈ [1, *N*]. The error terms are $m_{i,t},v_{i,t},s_{i,t}$ and $u_{i,t}$ and they are assumed to meet the conventional characteristics, namely that they are normally distributed, identically, independently, and devoid of autocorrelation and heteroskedasticity [35].

Descriptive statistical analysis, correlation, and IRF graphical representations were performed as the first phase in the analysis process. The second part of the investigation included a test of Granger causality test. This study initially performed unit root tests before doing the Granger causality test. Following that, lag selection criteria were used. The stability requirement was next examined, followed by the Granger causality analysis.

## 6 Empirical results and discussion

This section begins with a descriptive study of the variables of REC, NREC, and GDP, then moves on to a complete examination and discussion of the Granger causality findings from the test.

### 6.1 Descriptive analysis

The main stage of the analysis was to look at descriptive statistics for the research. “Appendixes B, C and D” compare the average REC, NREC, and GDP growth in the 1990–1999 and 2010–2019 decades for 152 countries.

**Fig. 2:**
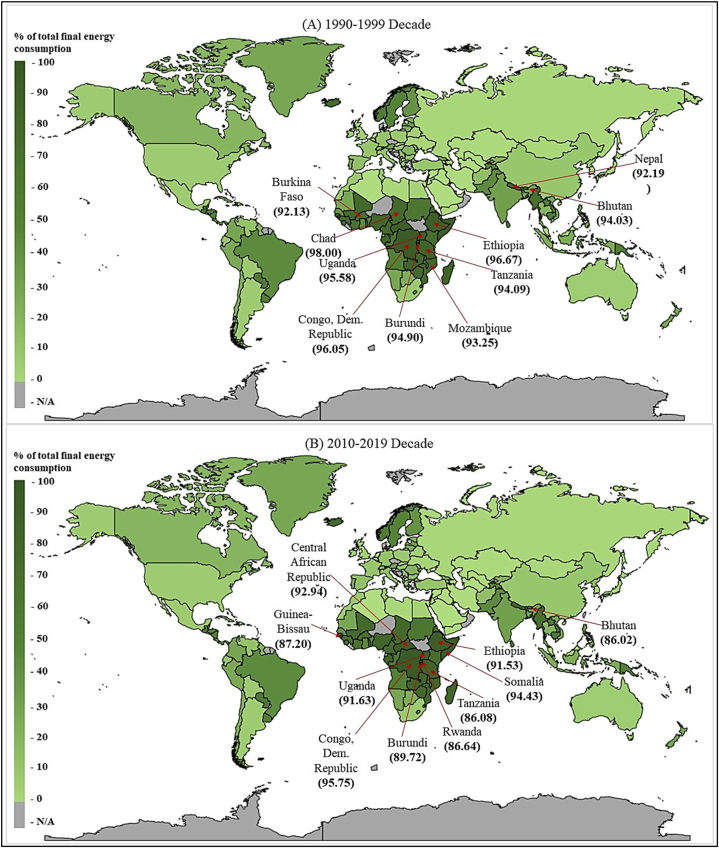
Average REC for 1990–1999 decade and 2010–2019 decade.

Source: Authors' Compilation.

The filled map for average REC for the 1990–1999 decade and the 2010–2019 decade is depicted in Fig. 2. In the 1990–1999-decade, Chad, Ethiopia, Congo Democratic Republic, Uganda, Burundi, Tanzania and Bhutan had the highest average REC, over 90 % of their total energy consumption. Furthermore, in the 2010–2019 decade, Congo Democratic Republic, Central African Republic, Uganda, Ethiopia, Burundi, Guinea-Bissau, Rwanda, Tanzania and Bhutan have the highest average REC from their total energy consumption. Based on the results, Kiribati, the United Kingdom, Belgium, Germany and Cyprus have managed to increase their REC by 905 %, 757 %, 633 %, 489 % and 424 % in the previous decade compared to the 1990–1999 decade.

Furthermore, Bulgaria, Ukraine, Netherlands, Ireland, Denmark, and the Slovak Republic have increased their REC from the total energy consumption. According to the results, it is clear that most of the LDCs are trying to use more renewable energy sources as they are rich in wind and solar power. However, their tremendous potential, solar and wind energy alone, will not meet the demands of LDCs. Hydroelectricity is also important, accounting for 50 % of all power produced in LDCs, and fossil fuels will keep playing an integral part in many situations, with a gradual move into less carbon-intensive alternatives such as gas from natural sources [60]. Most LDCs can reduce their indirect government governments by increasing REC because they use fossil fuels for their energy needs.

**Fig. 3:**
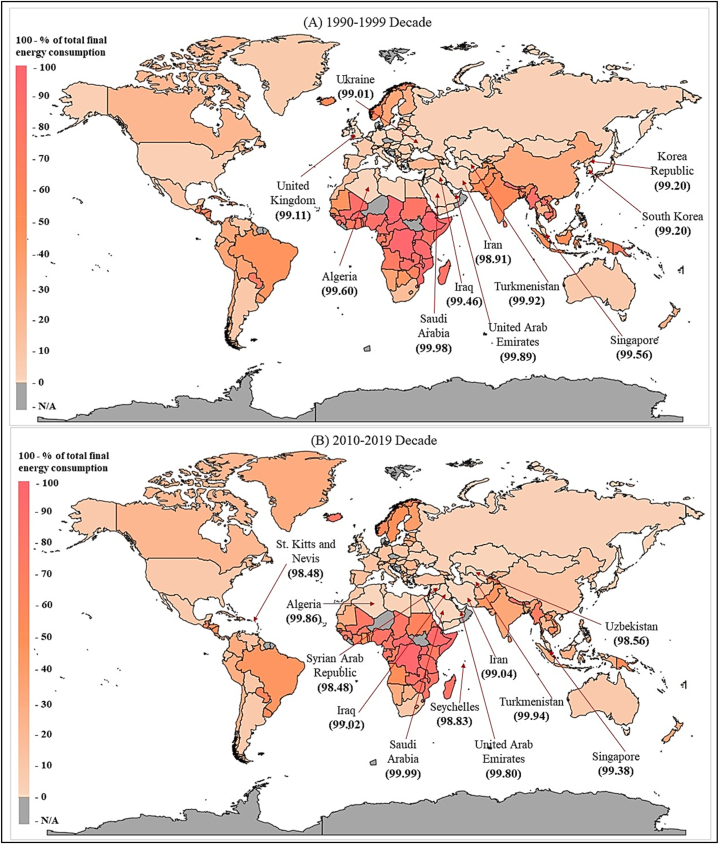
Average NREC for the 2010–2019 decade and 2010–2019 decade.

Source: Authors' Compilation.

The filled map for the average NREC for the 1990–1999 Decade and 2010–2019 decade is demonstrated in Fig. 3. This shows how the average NREC increased in the 2010–2019 decade compared to the 1990–1999 decade. In the 1990–1999-decade, Saudi Arabia, Turkmenistan, United Arab Emirates, Algeria, Singapore, Iraq, Korean Republic, United Kingdom, Ukraine and Iran had the highest NREC from their total energy consumption, over 90 %. Furthermore, in the 2010–2019-decade, Saudi Arabia, Turkmenistan, Algeria, United Arab Emirates, Trinidad and Tobago, Singapore, Iran, Iraq, Seychelles, States of Kitts and Nevis, Uzbekistan and Syria's highest NREC form their total final energy consumption. Based on the results, Chad, Benin, Equatorial Guinea, Burkina Faso, and Lao PDR countries have increased their NREC by 985 %, 370 %, 325 %, 235 % and 230 % accordingly in the previous decade compared to the 1990–1999 decade. Although Mozambique, Ghana, Ethiopia and Sierra Leone have also increased their NREC from the total energy consumption.

**Fig. 4:**
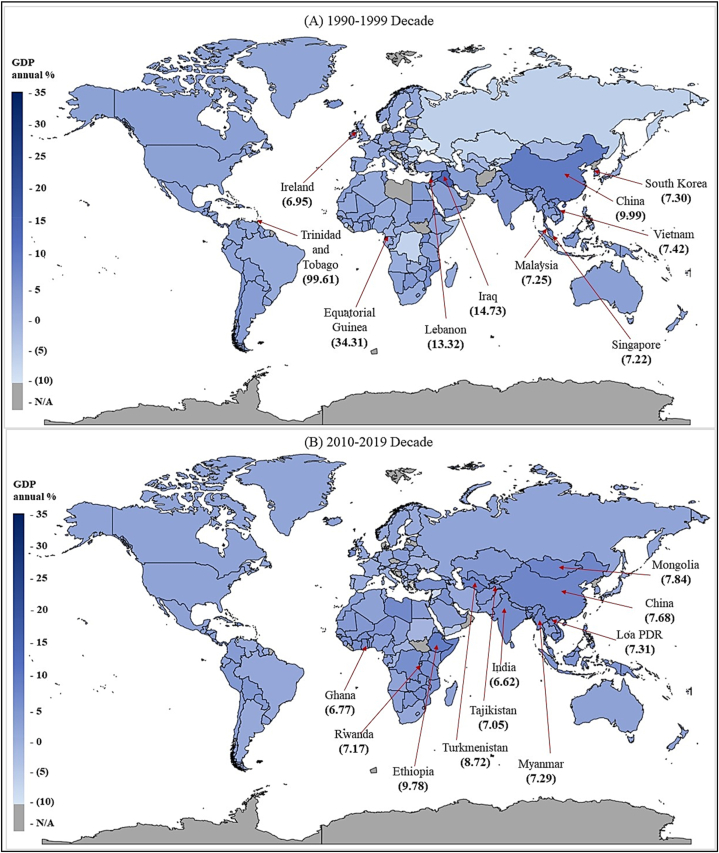
Average GDP for the 2010–2019 decade and 2010–2019 decade.

Source: Authors' Compilation.

The filled map for average GDP in the growth for the 1990–1999 decade and 2010–2019 decade is demonstrated in Fig. 4. In the 1990–1999-decade, Equatorial Guinea, Iraq, Lebanon, Cabo Verde, Korea, Vietnam, Korean Republic, Malaysia and Singapore had the highest GDP growth other compared to other nations. Further, 2010–2019 decade, Ethiopia, Turkmenistan, and Mongolia, China, Lao PDR, Myanmar and Rwanda have the highest GDP growth rates. Based on the results, Mongolia (2510 %), Uzbekistan and Cameroon (2272 %), Turkmenistan (644 %) and Haiti (589 %) have managed to increase their average GDP growth rate in the previous decade compared to the 1990–1999 decade.

Descriptive statistics data for REC, NREC, and economic growth for all nation categories is contained in “Appendix E”. Furthermore, “Appendix F” provides a detailed cross-country analysis for descriptive statistics.

**Fig. 5:**
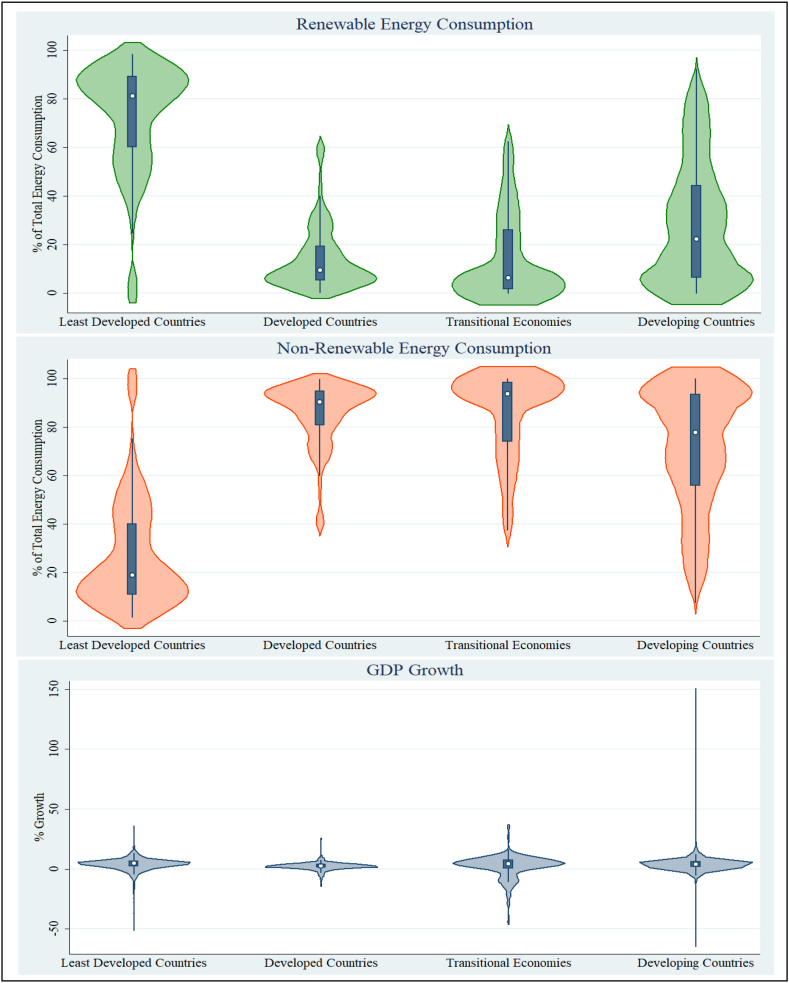
Violin Graph to monitor the REC, NREC and GDP.

Source: Authors' Compilation.

The violin plots of REC, NREC and GDP are shown in Fig. 5. Violin plots are comprehensively used to understand the distribution of data. The shape of the violins shows that the least developed countries use more renewable energy sources than other countries. Most of the REC percentage figures are near 80 % in the least developed countries, which is an excellent sign to design. Transitional economies and developing countries are using a higher proportion of NREC from their total primary energy consumption. According to the findings, prior research is employing renewable energy improves economic growth [5]. All these nations should increase the proportion of REC in their total energy usage to achieve better sustainable economic growth. Violin shapes of GDP growth rates show a similar form for all the nations implying an equal data distribution.

The correlation matrix for the factors under investigation in the present research is displayed in Table 2. For LDCs, developed, transitional economies and developing countries, there is a weak positive association between GDP and REC and a weak negative relationship between GDP and NREC. The worldwide correlation value of 0.082 reveals a modest positive link between GDP and REC, supporting the slight adverse association between GDP and NREC. Prior researchers have identified that increasing renewable energy usage impacts economic development in Lithuania, Slovenia and Hungary and confirms a linkage between GDP and REC in developed nations [61]. However, these findings vary with a study in the BRICS, which revealed that economic expansion favoured energy utilisation [62]. As per the results, relationships vary from country to country, and identifying the relationships between each variable will help policymakers undertake strategic decisions more accurately regarding renewable energy. Similar to the results in developing country category of current study, the authors of another research has been concluded that renewable energy is a solution for sustainable development in the face of India's economic growth direction [49]. Furthermore, while the current study has been identified a positive correlation between REC and GDP, another study has been identified fossil fuel energy use have a negative impact on environmental sustainability in South Africa, demonstrating a trade-off between economic growth and environmental quality [50].

**Table 2:** Correlation matrix of country categories.

Group	Variable	REC	NREC	GDP
Least-developed Countries	**REC**	1.0000***	−1.0000***	0.0762***
	**NREC**	−1.0000***	1.0000***	−0.0762***
	**GDP**	0.0762***	−0.0762***	1.0000***
Developed Countries	**REC**	1.0000***	−1.0000***	0.0582***
	**NREC**	−1.0000***	1.0000***	−0.0512***
	**GDP**	0.0582***	−0.0512***	1.0000***
Transitional Economies	**REC**	1.0000***	−1.0000***	0.0512***
	**NREC**	−1.0000***	1.0000***	−0.0512***
	**GDP**	0.0512***	−0.0512***	1.0000***
Developing Countries	**REC**	1.0000***	−1.0000***	0.0342***
	**NREC**	−1.0000***	1.0000***	−0.0342***
	**GDP**	0.0342***	−0.0342***	1.0000***
All countries	**REC**	1.0000***	−1.0000***	0.0829***
	**NREC**	−1.0000***	1.0000***	−0.0829***
	**GDP**	0.0829***	−0.0829***	1.0000***

Note: The symbol *** represents 1 % significance level.

### 6.2 Granger causality analysis

The LLC unit root test was used to identify whether or not the datasets were stationed using the Granger causality test.

All variables have significance at 1 % for each nation classification and globally, and the data series is stationary. The test findings in Table 3 indicate that at the 1 % significance level, the null hypothesis that the datasets include unit roots might be denied in preference for the alternative view that the datasets are stationary. Furthermore, “Appendix G″ presents a cross-country analysis from Unit Root for REC, NREC and GDP.

**Table 3:** LLC unit root test.

	DREC	DNREC	DGDP
Least-developed Countries	−13.0417***	−13.0417***	−23.6279***
Developed Countries	−8.5691***	−8.5692***	−19.6194***
Transitional economies	−12.2068***	−12.2070***	−14.3468***
Developing Countries	−15.3214***	−15.3216***	−31.7354***
Global view	−24.6919***	−24.6921***	−46.1213***

Note: The symbol *** represents 1 % significance level.

Throughout the lag determination step, the number of lags for every attribute was determined to identify the causal link between REC, NREC, and GDP. The Minimum Biofilm Inhibitory Concentration test was selected since it required a low value as the base of the lag determination criteria. For the remainder of the examination, Lag 1 was employed. The associated eigenvalue graph for each nation group is presented in “Appendix H”. In the plots, all eigenvalues lie within the unit round, indicating that pVAR meets the stability criteria. The Granger causality test was performed after determining if the panels had unit roots, selecting the lag lengths, and evaluating the stability requirement to evaluate the link between REC, NREC and GDP.

The country category-wise overall Granger causality results in Table 4 indicate an uni-directional causality running from REC to GDP and NREC to GDP in transitional economies. This implies energy consumption directly influences GDP growth in transitional economies. Based on the Granger causality results, LDCs, in developed and developing countries show no significant causal relationships between GDP and energy consumption. Our results differ from a study that found a bi-directional relationship between REC and GDP in developing countries [20]. Despite the effect seen in this study, some researchers identified that the value of the manufacturing sector which makes a substantial contribution to GDP and NREC, has a bi-directional causal relationship in developing countries [11].

**Table 4:** Overview of Granger causality Results for REC, NREC and GDP.

	DREC → DGDP	DGDP → DREC	DREC - DGDP
Least-developed Countries	0.4900	2.0990	**⇼**
Developed Countries	0.2650	0.5000	**⇼**
Transitional economies	0.7530	11.6320***	
Developing Countries	0.2340	0.0340	**⇼**
Global view	1.1380	0.0320	**⇼**
	**DNREC → DGDP**	**DGDP → DNREC**	**DNREC - DGDP**
Least-developed Countries	0.4900	2.0990	**⇼**
Developed Countries	0.2650	0.5000	**⇼**
Transitional economies	0.7530	11.6320***	
Developing Countries	0.2340	0.0340	**⇼**
Global view	1.1380	0.0320	**⇼**

Note: The symbols *, **, and *** represents 10 %, 5 %, and 1 % significance level, respectively. And the symbols **⇼,** and **→,** represents no causation relationship, and one-way-right direction causation relationship, respectively.

Moreover, different from this study's results, the bi-directional causality between REC and real GDP was founded in developed countries [19]. However, it has also been proved that an uni-directional causal relationship exists between NREC and GDP in transitional economies [35]. There is evidence for uni-directional and bi-directional relationships between REC, NREC and GDP in the existing literature.

Country-wise Granger causality results for LDCs between energy consumption and GDP growth are presented in Table 5. Based on the results, Bangladesh, Guinea-Bissau, Rwanda, and Uganda represent uni-directional causal relationship from GDP to REC and NREC. Also, Bhutan, Chad, Ethiopia, Nepal and Papua New Guinea indicate uni-directional relationship from REC and NREC to GDP. Moreover, A study based in Ghana found that REC has no Granger causality on economic growth [63], whereas the present research has also identified the same.

**Table 5:** Overview of Granger causality Results for REC, NREC and GDP in LDCs.

Least-developed Countries			
Country	DREC → DGDP	DGDP → DREC	DREC - DGDP
Bangladesh	2.7909***	1.2148	
Bhutan	0.2428	3.1147***	
Chad	1.0285	4.0563**	
Ethiopia	0.2388	4.2060**	
Guinea-Bissau	13.3810*	0.1972	
Nepal	0.1237	3.5973**	
Papua New Guinea	0.5436	3.1939**	
Rwanda	4.8911**	0.0509	
Uganda	8.889*	0.0256	
**Country**	**DNREC → DGDP**	**DGDP → DNREC**	**DNREC - DGDP**
Bangladesh	2.7909***	1.2148	
Bhutan	0.2428	3.1147***	
Chad	1.0285	4.0563**	
Ethiopia	0.2388	4.2060**	
Guinea-Bissau	13.3810*	0.1972	
Nepal	0.1237	3.5973**	
Papua New Guinea	0.5436	3.1939**	
Rwanda	4.8911**	0.0509	
Uganda	8.889*	0.0256	

Note: The symbols *, **, and *** represents 10 %, 5 %, and 1 % significance level, respectively. And the symbols **←** and **→** represents a one-way-left direction causation relationship, and a one-way-right direction causation relationship, respectively.

The country-wise Granger causality results for developed countries between energy consumption and GDP growth are presented in Table 6. Norway shows bi-directional causality between REC and NREC, and GDP. Bulgaria, France, Greece, Ireland, Italy, Netherlands, Poland and Spain indicate uni-directional causal relationship from GDP to REC and NREC. Similarly, Cyprus, Finland, Portugal and the Slovak Republic represent uni-directional causality from REC and NREC to GDP. According to the analysis, there is an uni-directional causal linkage between REC and NREC to GDP in Italy, and no causal relationship has been found in Germany and UK. However, another research has found different result to that, where there is long-run uni-directional causalities from renewable energy to economic expansion for Italy, Germany, and the UK, as well as short-run uni-directional causalities from economic development to REC in Italy and the UK [3]. Furthermore, some authors have revealed bi-directional causality between REC and GDP in Italy [25]. Previous literature proves causal relationships between the variables and developed nations can strategically identify the links between the variables.

**Table 6:** Overview of Granger causality Results for REC, NREC and GDP in Developed Countries.

Developed Countries			
Country	DREC → DGDP	DGDP → DREC	DREC - DGDP
Bulgaria	5.9032**	0.0386	
Cyprus	1.0412	6.8594*	
Finland	2.4153	7.4684*	
France	3.4957***	0.0002	
Greece	4.0050**	0.4542	
Ireland	5.7045**	1.4208	
Italy	6.7095**	0.8962	
Netherlands	15.3300*	0.6866	
Norway	3.5143***	12.8400*	
Poland	7.1986*	0.2888	
Portugal	1.6062	3.5690***	
Slovak Republic	0.1484	5.5285**	
Spain	3.9688**	1.6952	
**Country**	**DNREC → DGDP**	**DGDP → DNREC**	**DNREC - DGDP**
Bulgaria	5.9032**	0.0386	
Cyprus	1.0412	6.8594*	
Finland	2.4153	7.4684*	
France	3.4957***	0.0002	
Greece	4.0050**	0.4542	
Ireland	5.7045**	1.4208	
Italy	6.7095**	0.8962	
Netherlands	15.3300*	0.6866	
Norway	3.5143***	12.8400*	
Poland	7.1986*	0.2888	
Portugal	1.6062	3.5690***	
Slovak Republic	0.1484	5.5285**	
Spain	3.9688**	1.6952	

Note: The symbols *, **, and *** represents 10 %, 5 %, and 1 % significance level, respectively. And the symbols **↔ ←** and **→** represents a bi-directional, one-way-left direction causation relationship, and one-way-right direction causation relationship, respectively.

The country-wise Granger causality results for transitional economies between energy consumption and GDP growth is shown in Table 7. Albania and Armenia show uni-directional causal relationship from REC and NREC to GDP. Similarly, there are uni-direction relationships Kyrgyzstan Republic, Russian Federation and Turkmenistan from GDP to REC and NREC.

**Table 7:** Overview of Granger causality Results for REC, NREC and GDP in Transitional Economies.

Transitional economies			
Country	DREC → DGDP	DGDP → DREC	DREC - DGDP
Albania	1.4267	10.0910*	
Armenia	1.7295	3.8877**	
Kyrgyz Republic	8.3908*	1.1501	
Russian Federation	4.1215**	0.8126	
Turkmenistan	12.1430*	2.2027	
**Country**	**DNREC → DGDP**	**DGDP → DNREC**	**DNREC - DGDP**
Albania	1.4267	10.0910*	
Armenia	1.7295	3.8877**	
Kyrgyz Republic	8.3908*	1.1501	
Russian Federation	4.1215**	0.8126	
Turkmenistan	12.1430*	2.2027	

Note: The symbols *, **, and *** represents 10 %, 5 %, and 1 % significance level, respectively. And the symbols **←** and **→** represents a one-way-left direction causation relationship, and a one-way-right direction causation relationship, respectively.

The country-wise Granger causality results for developing countries between energy consumption and GDP growth are presented in Table 8. Fiji and Malaysia show a bi-directional causality between energy consumption and GDP growth. Furthermore, Botswana, Cuba, Ghana, India, Iraq, South Africa, the Syrian Arab Republic, and the United Arab Emirates indicate an uni-directional causal relationship from GDP to REC and NREC. Brazil, Lebanon, Mexico, Seychelles, Sri Lanka and Tunisia show an uni-directional causality from REC and NREC to GDP. A study by China identified a bi-directional causal relationship between REC and GDP, different from the results of [64]. Most existing literature shows uni-directional relationships between the variables for developing countries and developing nations and the interdependency between the variables to make decisions.

**Table 8:** Overview of Granger causality Results for REC, NREC and GDP in Developing Countries.

Developing Countries			
Country	DREC → DGDP	DGDP → DREC	DREC - DGDP
Botswana	2.7440***	0.0666	
Brazil	0.2311	6.4886**	
Cuba	5.3328**	1.0655	
Fiji	2.9181***	3.2710***	
Ghana	4.3782**	0.6589	
India	3.1687***	0.0318	
Iraq	5.8615**	2.3926	
Lebanon	0.8060	2.8002***	
Malaysia	4.1611**	3.7480 ***	
Mexico	0.1343	5.235**	
Seychelles	3.7054	0.7748***	
South Africa	4.2098**	0.0312	
Sri Lanka	0.2543	3.1648***	
Syrian Arab Republic	13.359*	0.3294	
Tunisia	0.0767	8.0173*	
United Arab Emirates	3.2195***	0.0745	
**Country**	**DNREC → DGDP**	**DGDP → DNREC**	**DNREC - DGDP**
Botswana	2.7440***	0.0666	
Brazil	0.2311	6.4886**	
Cuba	5.3328**	1.0655	
Fiji	2.9181***	3.2710***	
Ghana	4.3782**	0.6589	
India	3.1687***	0.0318	
Iraq	5.8615**	2.3926	
Lebanon	0.8060	2.8002***	
Malaysia	4.1611**	3.7480 ***	
Mexico	0.1343	5.2350**	
Seychelles	3.7054	0.7748***	
South Africa	4.2098**	0.0312	
Sri Lanka	0.2543	3.1648***	
Syrian Arab Republic	13.359*	0.3294	
Tunisia	0.0767	8.0173*	
United Arab Emirates	3.2195***	0.0745	

Note: The symbols *, **, and *** represents 10 %, 5 %, and 1 % significance level, respectively. And the symbols **↔ ←** and **→** represent the bi-directional, one-way-left direction causation relationship, and one-way-right direction causation relationship, respectively.

Furthermore, “Appendix I and J” show cross-country analysis from Granger causality between REC, NREC and GDP. Finally, the present research has run IRF graphs for each country category and is presented in “Appendix K and L”.

The choice of data may significantly influence results. Different studies use different datasets with different sample sizes, data frames and data quality. These differences can lead to changes in statistical relationships in the data sets and directly impact the final Granger results. Also, some assumptions are attached to the Granger causality methodology, such as lag lengths and model specifications. Granger causality results are directly influenced by the above factors in various studies. Due to these factors, the nature of the relationships, the strength of the relationships and the direction of causal relationships between different studies may vary according to their sensitivities.

## 7 Conclusion

Renewable energy sources are the leading solutions for climate change due to energy production activities. Most developed countries are now trying to revolutionise their energy policies towards solar, wind, hydro and geothermal power to achieve a better sustainable future. This study identified that the least developed countries and developing countries are using more renewable energy sources than other countries which in line with the previous literature. Renewable energy provides a wide range of ecological, economic, and social advantages, including reducing greenhouse gas emissions, promoting independence, reducing air pollution, discovering innovative energy solutions, and creating new employment possibilities. To remove obstacles and hasten the shift to a future powered by renewable energy, however, more study, technology development, and legislative assistance are required from every nation. In comparison to past researches, the current study divides the differences between countries under investigation as LDCs, developed, transitional economies, and developing countries to analyse the relationship between REC, NREC, and GDP growth. Previous studies did not take into account 152 countries separately under all four country categories together in one study. Furthermore, this study provides valuable insights for informed decision-making, accelerating the transition to a sustainable future by highlighting the potential of renewable energy sources for economic growth and climate change reduction.

This study does not address the social implications of using sources of renewable energy, such as job development, enhanced rural living quality, enhanced public wellness by decreasing pollutants, and improved literacy among experts and the general public. Future researchers may review the economic consequences of renewable energy by taking into account a variety of factors and variables, such as FDI, both private and public entities, advances in technology, research and development, misconduct, and national financial stability.

## 8 Policy implications

Non-renewable energy sources such as fossil fuel, gasoline, and nuclear energy have significantly impacted the world from the environmental side. Due to the limited availability, all these non-renewable energy sources will be exhausted soon. Most countries are implementing renewable energy and sustainability-related initiatives as their principal investments. Based on the results, developed countries like the Netherlands and Denmark have achieved sustainable economic growth by increasing their average REC through the decades. Most of the developed nations can invest and research primary energy sources of each country and implement their own energy co-option patterns and development strategies compared to other nations due to their resource availability.

Most Asian LDCs and developing countries show uni-directional causality between energy consumption and GDP growth. Also, LDCs are using more renewable energy sources than other nations, which average 79 % of the total energy consumption. Those countries should evaluate their energy efficiency and wastage levels to achieve a greener and more sustainable future. Also, LDCs and developing countries can attract foreign direct investments the sustainable energy production strategies. Furthermore, African countries and countries near the equator, which typically receive considerable sunlight, can easily invest more in solar energy, which is cost-effective and convenient.

Transitional economies have the lowest average REC, which is fifteen per cent, and results show bi-directional causality between energy consumption and GDP Growth. This indicates each variable will have a direct impact on the other variable. Transitional economies should implement and invest more in renewable energy sources for better sustainable economic growth. Moreover, policymakers should focus on both variables when making decisions because the results show that energy consumption and economic growth are interconnected.

Implementing worldwide energy efficiency standards, lowering fossil fuel consumption, and enacting regulatory measures are efficient environmental strategies for mitigating environmental hazards while promoting economic growth. Furthermore, prioritising infrastructure investment, implementing carbon price, and raising societal awareness are equally critical.

Ultimately, every nation has its energy sources that produce energy. Everyone is responsible for using clean energy sources as much as possible because using cleaner energy sources will have a genuine and a positive impact on the world.

## Data availability statement

All data used in this study are given in the supplemental information file and publicly accessible through the World Bank open database.

## CRediT authorship contribution statement

**Nishitha Perera:** Writing – original draft, Visualization, Validation, Software, Methodology, Formal analysis, Data curation, Conceptualization. **Hasara Dissanayake:** Writing – original draft, Visualization, Validation, Software, Methodology, Data curation, Conceptualization. **Diruni Samson:** Writing – original draft, Visualization, Validation, Data curation. **Sajani Abeykoon:** Writing – original draft, Visualization, Validation, Data curation. **Ruwan Jayathilaka:** Writing – review & editing, Writing – original draft, Validation, Supervision, Methodology, Conceptualization. **Maneka Jayasinghe:** Writing – review & editing, Writing – original draft, Supervision. **Shanta Yapa:** Writing – original draft, Supervision.

## Declaration of competing interest

The authors declare that they have no known competing financial interests or personal relationships that could have appeared to influence the work reported in this paper.

## Footnotes

1 Dutch disease is an economic phrase describing the unfavorable effects that can result from a sharp increase in a country's currency value.
