# Supplementary material for: The interconnectedness of energy consumption with economic growth: A granger causality analysis
Source: Heliyon. 2024 Aug 28;10(17):e36709. doi: 10.1016/j.heliyon.2024.e36709 (PMC11402754; doi:10.1016/j.heliyon.2024.e36709)
Supplement: Multimedia component 2 [file mmc2.docx]

**Appendix B. Average Renewable Energy Consumption**

| **Least-developed Countries** | | | |
| --- | --- | --- | --- |
| **Country** | **1990-1999** | **2010-2019** | **Increase / Decrease in REC** |
| Angola | 73.141 | 51.439 | ▼29.67% |
| Bangladesh | 66.269 | 33.081 | ▼50.08% |
| Benin | 88.950 | 48.042 | ▼45.99% |
| Bhutan | 94.031 | 86.019 | ▼8.52% |
| Burkina Faso | 92.135 | 73.575 | ▼20.14% |
| Burundi | 94.902 | 89.717 | ▼5.46% |
| Central African Republic | 91.935 | 92.937 | ▲1.09% |
| Chad | 98.005 | 78.330 | ▼20.08% |
| Comoros | 54.039 | 62.165 | ▲15.04% |
| Congo, Demographic Republic | 96.055 | 95.745 | ▼0.32% |
| Congo Republic | 72.451 | 64.892 | ▼10.43% |
| Ethiopia | 96.665 | 91.532 | ▼5.31% |
| Gambia | 59.944 | 52.613 | ▼12.23% |
| Guinea | 87.192 | 74.272 | ▼14.82% |
| Guinea-Bissau | 88.562 | 87.199 | ▼1.54% |
| Haiti | 82.634 | 77.942 | ▼5.68% |
| Kiribati | 4.481 | 45.068 | ▲905.86% |
| Lao PDR | 86.748 | 56.186 | ▼35.23% |
| Lesotho | 51.681 | 43.149 | ▼16.51% |
| Madagascar | 82.126 | 82.818 | ▲0.84% |
| Malawi | 81.961 | 78.542 | ▼4.17% |
| Mali | 87.511 | 78.435 | ▼10.37% |
| Mauritania | 45.451 | 30.133 | ▼33.70% |
| Mozambique | 93.247 | 81.721 | ▼12.36% |
| Myanmar | 87.025 | 71.286 | ▼18.09% |
| Nepal | 92.190 | 82.136 | ▼10.91% |
| Nigeria | 86.865 | 82.866 | ▼4.60% |
| Papua New Guinea | 70.211 | 55.073 | ▼21.56% |
| Rwanda | 85.355 | 86.638 | ▲1.50% |
| Senegal | 52.451 | 41.945 | ▼20.03% |
| Sierra Leone | 90.500 | 76.910 | ▼15.02% |
| Solomon Islands | 61.107 | 46.357 | ▼24.14% |
| Sudan | 80.031 | 62.588 | ▼21.80% |
| Tanzania | 94.089 | 86.081 | ▼8.51% |
| Togo | 80.467 | 75.589 | ▼6.06% |
| Uganda | 95.577 | 91.626 | ▼4.13% |
| Yemen Republic | 1.586 | 1.826 | ▲15.16% |
| Zambia | 86.128 | 84.366 | ▼2.05% |
| **Developed Countries** | | | |
| **Country** | **1990-1999** | **2010-2019** | **Increase / Decrease in REC** |
| Andorra | 14.312 | 18.934 | ▲32.30% |
| Australia | 8.391 | 9.152 | ▲9.06% |
| Austria | 25.319 | 33.875 | ▲33.79% |
| Belgium | 1.186 | 8.698 | ▲633.34% |
| Bulgaria | 3.468 | 17.013 | ▲390.61% |
| Cyprus | 1.876 | 9.838 | ▲424.38% |
| Denmark | 7.735 | 30.164 | ▲289.96% |
| Finland | 26.849 | 40.710 | ▲51.62% |
| France | 10.514 | 13.405 | ▲27.49% |
| Germany | 2.423 | 14.274 | ▲489.07% |
| Greece | 7.962 | 15.657 | ▲96.64% |
| Hungary | 4.931 | 15.027 | ▲204.74% |
| Ireland | 2.104 | 8.556 | ▲306.60% |
| Italy | 4.713 | 15.597 | ▲230.92% |
| Japan | 4.119 | 5.917 | ▲43.67% |
| Luxembourg | 3.428 | 9.462 | ▲176.00% |
| Netherlands | 1.336 | 5.751 | ▲330.52% |
| New Zealand | 29.270 | 31.047 | ▲6.07% |
| North America | 6.526 | 10.280 | ▲57.54% |
| Norway | 59.850 | 58.712 | ▼1.90% |
| Poland | 5.032 | 11.161 | ▲121.79% |
| Portugal | 23.954 | 27.749 | ▲15.84% |
| Romania | 9.903 | 23.206 | ▲134.34% |
| Slovak Republic | 3.485 | 12.303 | ▲253.06% |
| Spain | 9.111 | 16.340 | ▲79.34% |
| Sweden | 33.683 | 49.333 | ▲46.46% |
| Switzerland | 17.826 | 22.903 | ▲28.48% |
| United Kingdom | 0.891 | 7.640 | ▲757.69% |
| United States | 4.607 | 9.178 | ▲99.24% |
| **Transitional economies** | | | |
| **Country** | **1990-1999** | **2010-2019** | **Increase / Decrease in REC** |
| Albania | 45.884 | 38.572 | ▼15.94% |
| Armenia | 9.141 | 9.570 | ▲4.69% |
| Azerbaijan | 1.597 | 2.521 | ▲57.89% |
| Belarus | 2.272 | 7.164 | ▲215.26% |
| Georgia | 32.487 | 30.085 | ▼7.39% |
| Kazakhstan | 1.525 | 1.598 | ▲4.76% |
| Kyrgyz Republic | 22.550 | 24.626 | ▲9.20% |
| North Macedonia | 13.493 | 20.301 | ▲50.45% |
| Russian Federation | 3.812 | 3.293 | ▼13.62% |
| Tajikistan | 49.484 | 48.761 | ▼1.46% |
| Turkmenistan | 0.080 | 0.057 | ▼28.83% |
| Ukraine | 0.988 | 4.594 | ▲365.04% |
| Uzbekistan | 1.402 | 1.436 | ▲2.43% |
| **Developing Countries** | | | |
| **Country** | **1990-1999** | **2010-2019** | **Increase / Decrease in REC** |
| Algeria | 0.401 | 0.145 | ▼63.83% |
| Argentina | 10.205 | 9.534 | ▼6.58% |
| Barbados | 15.883 | 4.678 | ▼70.55% |
| Belize | 36.104 | 36.952 | ▲2.35% |
| Bolivia | 34.783 | 10.357 | ▼70.22% |
| Botswana | 44.763 | 26.627 | ▼40.52% |
| Brazil | 46.640 | 44.852 | ▼3.83% |
| Cabo Verde | 37.082 | 23.727 | ▼36.01% |
| Cameroon | 84.126 | 78.496 | ▼6.69% |
| Chile | 33.566 | 26.777 | ▼20.23% |
| China | 31.364 | 12.455 | ▼60.29% |
| Colombia | 32.401 | 30.854 | ▼4.77% |
| Costa Rica | 36.113 | 37.469 | ▲3.75% |
| Cote d'Ivoire | 72.933 | 69.004 | ▼5.39% |
| Cuba | 42.206 | 20.359 | ▼51.76% |
| Dominica | 15.185 | 10.282 | ▼32.29% |
| Dominican Republic | 23.301 | 16.096 | ▼30.92% |
| Ecuador | 20.707 | 14.110 | ▼31.86% |
| Egypt Arab Republic | 8.777 | 5.407 | ▼38.40% |
| El Salvador | 57.743 | 25.323 | ▼56.15% |
| Equatorial Guinea | 77.592 | 4.716 | ▼93.92% |
| Eswatini | 72.355 | 69.654 | ▼3.73% |
| Fiji | 57.511 | 29.660 | ▼48.43% |
| Gabon | 72.933 | 83.738 | ▲14.82% |
| Ghana | 78.973 | 45.933 | ▼41.84% |
| Grenada | 8.199 | 11.037 | ▲34.62% |
| Guatemala | 68.274 | 65.433 | ▼4.16% |
| Guyana | 38.646 | 23.826 | ▼38.35% |
| Honduras | 64.637 | 50.814 | ▼21.39% |
| India | 55.120 | 33.920 | ▼38.46% |
| Indonesia | 52.081 | 27.375 | ▼47.44% |
| Iran Islamic Republic | 1.091 | 0.958 | ▼12.16% |
| Iraq | 0.544 | 0.982 | ▲80.51% |
| Jamaica | 9.371 | 10.639 | ▲13.54% |
| Jordan | 2.398 | 4.357 | ▲81.70% |
| Kenya | 79.373 | 73.903 | ▼6.89% |
| Korea Republic | 0.797 | 2.374 | ▲197.76% |
| Lebanon | 6.281 | 4.991 | ▼20.53% |
| Malaysia | 9.107 | 3.590 | ▼60.58% |
| Marshall Islands | 19.415 | 11.947 | ▼38.46% |
| Mauritius | 37.318 | 10.758 | ▼71.17% |
| Mexico | 13.022 | 9.516 | ▼26.92% |
| Micronesia Federal States | 1.462 | 1.570 | ▲7.42% |
| Mongolia | 2.916 | 3.453 | ▲18.43% |
| Morocco | 17.669 | 11.381 | ▼35.59% |
| Namibia | 36.590 | 30.448 | ▼16.78% |
| Nicaragua | 64.948 | 51.891 | ▼20.10% |
| Pakistan | 53.899 | 45.503 | ▼15.58% |
| Panama | 37.964 | 21.307 | ▼43.88% |
| Paraguay | 71.964 | 61.704 | ▼14.26% |
| Peru | 34.977 | 28.774 | ▼17.73% |
| Philippines | 41.903 | 30.684 | ▼26.77% |
| Samoa | 45.493 | 38.657 | ▼15.03% |
| Saudi Arabia | 0.021 | 0.014 | ▼32.31% |
| Seychelles | 2.547 | 1.173 | ▼53.95% |
| Singapore | 0.441 | 0.618 | ▲40.03% |
| South Africa | 17.776 | 10.530 | ▼40.76% |
| Sri Lanka | 70.375 | 55.344 | ▼21.36% |
| St. Kitts and Nevis | 34.973 | 1.430 | ▼95.91% |
| St. Lucia | 3.586 | 11.524 | ▲221.39% |
| St. Vincent and the Grenadines | 10.353 | 4.923 | ▼52.45% |
| Syrian Arab Republic | 1.951 | 1.523 | ▼21.95% |
| Thailand | 25.685 | 23.196 | ▼9.69% |
| Tonga | 1.311 | 1.606 | ▲22.51% |
| Trinidad and Tobago | 1.130 | 0.394 | ▼65.12% |
| Tunisia | 14.371 | 12.571 | ▼12.52% |
| Turkey | 22.736 | 12.915 | ▼43.19% |
| United Arab Emirates | 0.114 | 0.199 | ▲74.10% |
| Uruguay | 40.347 | 56.154 | ▲39.18% |
| Vanuatu | 26.822 | 35.393 | ▲31.96% |
| Vietnam | 67.307 | 30.108 | ▼55.27% |
| Zimbabwe | 65.847 | 80.509 | ▲22.27% |
