# Supplementary material for: The interconnectedness of energy consumption with economic growth: A granger causality analysis
Source: Heliyon. 2024 Aug 28;10(17):e36709. doi: 10.1016/j.heliyon.2024.e36709 (PMC11402754; doi:10.1016/j.heliyon.2024.e36709)
Supplement: Multimedia component 3 [file mmc3.docx]

**Appendix C. Average Non-renewable Energy Consumption**

| **Least-developed Countries** | | | |
| --- | --- | --- | --- |
| **Country** | **1990-1999** | **2010-2019** | **Increase / Decrease in NREC** |
| Angola | 26.859 | 48.561 | ▲80.80% |
| Bangladesh | 33.731 | 66.919 | ▲98.39% |
| Benin | 11.050 | 51.958 | ▲370.22% |
| Bhutan | 5.969 | 13.981 | ▲134.24% |
| Burkina Faso | 7.865 | 26.425 | ▲235.96% |
| Burundi | 5.098 | 10.283 | ▲101.69% |
| Central African Republic | 8.065 | 7.063 | ▼12.42% |
| Chad | 1.995 | 21.670 | ▲985.96% |
| Comoros | 45.961 | 37.835 | ▼17.68% |
| Congo, Demographic Republic | 3.945 | 4.255 | ▲7.85% |
| Congo Republic | 27.549 | 35.108 | ▲27.44% |
| Ethiopia | 3.335 | 8.468 | ▲153.93% |
| Gambia | 40.056 | 47.387 | ▲18.30% |
| Guinea | 12.808 | 25.728 | ▲100.87% |
| Guinea-Bissau | 11.438 | 12.801 | ▲11.92% |
| Haiti | 17.366 | 22.058 | ▲27.02% |
| Kiribati | 95.519 | 54.932 | ▼42.49% |
| Lao PDR | 13.252 | 43.814 | ▲230.63% |
| Lesotho | 48.319 | 56.851 | ▲17.66% |
| Madagascar | 17.874 | 17.182 | ▼3.87% |
| Malawi | 18.039 | 21.458 | ▲18.96% |
| Mali | 12.489 | 21.565 | ▲72.67% |
| Mauritania | 54.549 | 69.867 | ▲28.08% |
| Mozambique | 6.753 | 18.279 | ▲170.66% |
| Myanmar | 12.975 | 28.714 | ▲121.31% |
| Nepal | 7.810 | 17.864 | ▲128.72% |
| Nigeria | 13.135 | 17.134 | ▲30.45% |
| Papua New Guinea | 29.789 | 44.927 | ▲50.82% |
| Rwanda | 14.645 | 13.362 | ▼8.76% |
| Senegal | 47.549 | 58.055 | ▲22.09% |
| Sierra Leone | 9.500 | 23.090 | ▲143.06% |
| Solomon Islands | 38.893 | 53.643 | ▲37.92% |
| Sudan | 19.969 | 37.412 | ▲87.35% |
| Tanzania | 5.911 | 13.919 | ▲135.48% |
| Togo | 19.533 | 24.411 | ▲24.97% |
| Uganda | 4.423 | 8.374 | ▲89.35% |
| Yemen Republic | 98.414 | 98.174 | ▼0.24% |
| Zambia | 13.872 | 15.634 | ▲12.71% |
| **Developed Countries** | | | |
| **Country** | **1990-1999** | **2010-2019** | **Increase / Decrease in NREC** |
| Andorra | 85.688 | 81.066 | ▼5.39% |
| Australia | 91.609 | 90.848 | ▼0.83% |
| Austria | 74.681 | 66.125 | ▼11.46% |
| Belgium | 98.814 | 91.302 | ▼7.60% |
| Bulgaria | 96.532 | 82.987 | ▼14.03% |
| Cyprus | 98.124 | 90.162 | ▼8.11% |
| Denmark | 92.265 | 69.836 | ▼24.31% |
| Finland | 73.151 | 59.290 | ▼18.95% |
| France | 89.486 | 86.595 | ▼3.23% |
| Germany | 97.577 | 85.726 | ▼12.15% |
| Greece | 92.038 | 84.343 | ▼8.36% |
| Hungary | 95.069 | 84.973 | ▼10.62% |
| Ireland | 97.896 | 91.444 | ▼6.59% |
| Italy | 95.287 | 84.403 | ▼11.42% |
| Japan | 95.881 | 94.083 | ▼1.88% |
| Luxembourg | 96.572 | 90.538 | ▼6.25% |
| Netherlands | 98.664 | 94.249 | ▼4.47% |
| New Zealand | 70.730 | 68.953 | ▼2.51% |
| North America | 93.474 | 89.720 | ▼4.02% |
| Norway | 40.150 | 41.288 | ▲2.83% |
| Poland | 94.968 | 88.839 | ▼6.45% |
| Portugal | 76.046 | 72.251 | ▼4.99% |
| Romania | 90.097 | 76.794 | ▼14.77% |
| Slovak Republic | 96.515 | 87.697 | ▼9.14% |
| Spain | 90.889 | 83.660 | ▼7.95% |
| Sweden | 66.317 | 50.667 | ▼23.60% |
| Switzerland | 82.174 | 77.097 | ▼6.18% |
| United Kingdom | 99.109 | 92.360 | ▼6.81% |
| United States | 95.393 | 90.822 | ▼4.79% |
| **Transitional economies** | | | |
| **Country** | **1990-1999** | **2010-2019** | **Increase / Decrease in NREC** |
| Albania | 54.116 | 61.428 | ▲13.51% |
| Armenia | 90.859 | 90.430 | ▼0.47% |
| Azerbaijan | 98.403 | 97.479 | ▼0.94% |
| Belarus | 97.728 | 92.836 | ▼5.01% |
| Georgia | 67.513 | 69.915 | ▲3.56% |
| Kazakhstan | 98.475 | 98.402 | ▼0.07% |
| Kyrgyz Republic | 77.450 | 75.374 | ▼2.68% |
| North Macedonia | 86.507 | 79.699 | ▼7.87% |
| Russian Federation | 96.188 | 96.707 | ▲0.54% |
| Tajikistan | 50.516 | 51.239 | ▲1.43% |
| Turkmenistan | 99.920 | 99.943 | ▲0.02% |
| Ukraine | 99.012 | 95.406 | ▼3.64% |
| Uzbekistan | 98.598 | 98.564 | ▼0.03% |
| **Developing Countries** | | | |
| **Country** | **1990-1999** | **2010-2019** | **Increase / Decrease in NREC** |
| Algeria | 99.599 | 99.855 | ▲0.26% |
| Argentina | 89.795 | 90.466 | ▲0.75% |
| Barbados | 84.117 | 95.322 | ▲13.32% |
| Belize | 63.896 | 63.048 | ▼1.33% |
| Bolivia | 65.217 | 89.643 | ▲37.45% |
| Botswana | 55.237 | 73.373 | ▲32.83% |
| Brazil | 53.360 | 55.148 | ▲3.35% |
| Cabo Verde | 62.918 | 76.273 | ▲21.23% |
| Cameroon | 15.874 | 21.504 | ▲35.47% |
| Chile | 66.434 | 73.223 | ▲10.22% |
| China | 68.636 | 87.545 | ▲27.55% |
| Colombia | 67.599 | 69.146 | ▲2.29% |
| Costa Rica | 63.887 | 62.531 | ▼2.12% |
| Cote d'Ivoire | 27.067 | 30.996 | ▲14.51% |
| Cuba | 57.794 | 79.641 | ▲37.80% |
| Dominica | 84.815 | 89.718 | ▲5.78% |
| Dominican Republic | 76.699 | 83.904 | ▲9.39% |
| Ecuador | 79.293 | 85.890 | ▲8.32% |
| Egypt Arab Republic | 91.223 | 94.593 | ▲3.69% |
| El Salvador | 42.257 | 74.677 | ▲76.72% |
| Equatorial Guinea | 22.408 | 95.284 | ▲325.23% |
| Eswatini | 27.645 | 30.346 | ▲9.77% |
| Fiji | 42.489 | 70.340 | ▲65.55% |
| Gabon | 27.067 | 16.262 | ▼39.92% |
| Ghana | 21.027 | 54.067 | ▲157.13% |
| Grenada | 91.801 | 88.963 | ▼3.09% |
| Guatemala | 31.726 | 34.567 | ▲8.95% |
| Guyana | 61.354 | 76.174 | ▲24.15% |
| Honduras | 35.363 | 49.186 | ▲39.09% |
| India | 44.880 | 66.080 | ▲47.24% |
| Indonesia | 47.919 | 72.625 | ▲51.56% |
| Iran Islamic Republic | 98.909 | 99.042 | ▲0.13% |
| Iraq | 99.456 | 99.018 | ▼0.44% |
| Jamaica | 90.629 | 89.361 | ▼1.40% |
| Jordan | 97.602 | 95.643 | ▼2.01% |
| Kenya | 20.627 | 26.097 | ▲26.52% |
| Korea Republic | 99.203 | 97.626 | ▼1.59% |
| Lebanon | 93.719 | 95.009 | ▲1.38% |
| Malaysia | 90.893 | 96.410 | ▲6.07% |
| Marshall Islands | 80.585 | 88.053 | ▲9.27% |
| Mauritius | 62.682 | 89.242 | ▲42.37% |
| Mexico | 86.978 | 90.484 | ▲4.03% |
| Micronesia Federal States | 98.538 | 98.430 | ▼0.11% |
| Mongolia | 97.084 | 96.547 | ▼0.55% |
| Morocco | 82.331 | 88.619 | ▲7.64% |
| Namibia | 63.410 | 69.552 | ▲9.69% |
| Nicaragua | 35.052 | 48.109 | ▲37.25% |
| Pakistan | 46.101 | 54.497 | ▲18.21% |
| Panama | 62.036 | 78.693 | ▲26.85% |
| Paraguay | 28.036 | 38.296 | ▲36.60% |
| Peru | 65.023 | 71.226 | ▲9.54% |
| Philippines | 58.097 | 69.316 | ▲19.31% |
| Samoa | 54.507 | 61.343 | ▲12.54% |
| Saudi Arabia | 99.979 | 99.986 | ▲0.01% |
| Seychelles | 97.453 | 98.827 | ▲1.41% |
| Singapore | 99.559 | 99.382 | ▼0.18% |
| South Africa | 82.224 | 89.470 | ▲8.81% |
| Sri Lanka | 29.625 | 44.656 | ▲50.74% |
| St. Kitts and Nevis | 65.027 | 98.570 | ▲51.58% |
| St. Lucia | 96.414 | 88.476 | ▼8.23% |
| St. Vincent and the Grenadines | 89.647 | 95.077 | ▲6.06% |
| Syrian Arab Republic | 98.049 | 98.477 | ▲0.44% |
| Thailand | 74.315 | 76.804 | ▲3.35% |
| Tonga | 98.689 | 98.394 | ▼0.30% |
| Trinidad and Tobago | 98.870 | 99.606 | ▲0.74% |
| Tunisia | 85.629 | 87.429 | ▲2.10% |
| Turkey | 77.264 | 87.085 | ▲12.71% |
| United Arab Emirates | 99.886 | 99.801 | ▼0.08% |
| Uruguay | 59.653 | 43.846 | ▼26.50% |
| Vanuatu | 73.178 | 64.607 | ▼11.71% |
| Vietnam | 32.693 | 69.892 | ▲113.78% |
| Zimbabwe | 34.153 | 19.491 | ▼42.93% |
