# Supplementary material for: The interconnectedness of energy consumption with economic growth: A granger causality analysis
Source: Heliyon. 2024 Aug 28;10(17):e36709. doi: 10.1016/j.heliyon.2024.e36709 (PMC11402754; doi:10.1016/j.heliyon.2024.e36709)
Supplement: Multimedia component 4 [file mmc4.docx]

**Appendix D. Average GDP Growth**

| **Least-developed Countries** | | | |  |
| --- | --- | --- | --- | --- |
| **Country** | **1990-1999** | **2010-2019** | **Increase / Decrease in GDP** | |
| Angola | 1.175 | 2.283 | ▲48.54% | |
| Bangladesh | 4.713 | 6.609 | ▲28.68% | |
| Benin | 4.942 | 4.779 | ▼3.42% | |
| Bhutan | 5.343 | 6.117 | ▲12.67% | |
| Burkina Faso | 5.123 | 6.002 | ▲14.64% | |
| Burundi | -1.433 | 2.219 | ▲164.59% | |
| Central African Republic | 1.291 | -0.193 | ▼769.82% | |
| Chad | 2.216 | 3.426 | ▲35.32% | |
| Comoros | 1.551 | 3.135 | ▲50.52% | |
| Congo Demographic Republic | -5.469 | 6.227 | ▲187.83% | |
| Congo Republic | 0.834 | 0.448 | ▼86.18% | |
| Ethiopia | 2.664 | 9.779 | ▲72.76% | |
| Gambia | 3.112 | 2.877 | ▼8.17% | |
| Guinea | 4.093 | 6.090 | ▲32.80% | |
| Guinea-Bissau | 1.303 | 3.930 | ▲66.86% | |
| Haiti | 0.187 | 1.286 | ▲85.49% | |
| Kiribati | 1.270 | 2.272 | ▲44.12% | |
| Lao PDR | 6.274 | 7.312 | ▲14.20% | |
| Lesotho | 4.406 | 2.447 | ▼80.06% | |
| Madagascar | 1.620 | 2.951 | ▲45.09% | |
| Malawi | 4.132 | 4.364 | ▲5.32% | |
| Mali | 3.905 | 4.390 | ▲11.04% | |
| Mauritania | 2.275 | 4.288 | ▲46.95% | |
| Mozambique | 6.333 | 5.559 | ▼13.92% | |
| Myanmar | 5.751 | 7.286 | ▲21.08% | |
| Nepal | 4.845 | 5.011 | ▲3.31% | |
| Nigeria | 2.314 | 3.650 | ▲36.59% | |
| Papua New Guinea | 4.313 | 5.307 | ▲18.72% | |
| Rwanda | 1.763 | 7.167 | ▲75.40% | |
| Senegal | 2.726 | 4.832 | ▲43.58% | |
| Sierra Leone | -2.620 | 5.048 | ▲151.90% | |
| Solomon Islands | 4.459 | 4.191 | ▼6.39% | |
| Sudan | 5.184 | -0.852 | ▼708.71% | |
| Tanzania | 3.269 | 6.308 | ▲48.18% | |
| Togo | 2.620 | 5.716 | ▲54.16% | |
| Uganda | 6.883 | 5.340 | ▼28.89% | |
| Yemen Republic | 5.672 | -3.827 | ▼248.20% | |
| Zambia | 1.312 | 4.889 | ▲73.17% | |
| **Developed Countries** | | | |  |
| **Country** | **1990-1999** | **2010-2019** | **Increase / Decrease in GDP** | |
| Andorra | 3.238 | 0.109 | ▼2860.59% | |
| Australia | 3.275 | 2.592 | ▼26.38% | |
| Austria | 2.706 | 1.538 | ▼75.91% | |
| Belgium | 2.177 | 1.622 | ▼34.23% | |
| Bulgaria | -3.516 | 2.076 | ▲269.39% | |
| Cyprus | 4.750 | 1.725 | ▼175.34% | |
| Denmark | 2.452 | 1.850 | ▼32.55% | |
| Finland | 1.884 | 1.198 | ▼57.31% | |
| France | 2.017 | 1.420 | ▼42.07% | |
| Germany | 2.174 | 1.971 | ▼10.30% | |
| Greece | 2.187 | -2.087 | ▼204.79% | |
| Hungary | 1.136 | 2.779 | ▲59.12% | |
| Ireland | 6.949 | 6.286 | ▼10.55% | |
| Italy | 1.508 | 0.276 | ▼446.31% | |
| Japan | 1.503 | 1.213 | ▼23.90% | |
| Luxembourg | 4.691 | 2.610 | ▼79.69% | |
| Netherlands | 3.319 | 1.454 | ▼128.34% | |
| New Zealand | 2.830 | 2.917 | ▲2.98% | |
| North America | 3.207 | 2.249 | ▼42.59% | |
| Norway | 3.560 | 1.462 | ▼143.54% | |
| Poland | 3.723 | 3.663 | ▼1.63% | |
| Portugal | 2.923 | 0.870 | ▼235.86% | |
| Romania | -1.534 | 3.106 | ▲149.37% | |
| Slovak Republic | 4.582 | 3.020 | ▼51.76% | |
| Spain | 2.661 | 1.055 | ▼152.21% | |
| Sweden | 1.746 | 2.547 | ▲31.45% | |
| Switzerland | 1.191 | 2.009 | ▲40.70% | |
| United Kingdom | 2.238 | 2.028 | ▼10.34% | |
| United States | 3.226 | 2.251 | ▼43.34% | |
| **Transitional economies** | | | | |
| **Country** | **1990-1999** | **2010-2019** | **Increase / Decrease in GDP** | |
| Albania | 0.632 | 2.589 | ▲75.59% | |
| Armenia | -4.021 | 4.470 | ▲211.16% | |
| Azerbaijan | -6.898 | 1.807 | ▲126.19% | |
| Belarus | -2.260 | 1.832 | ▲181.06% | |
| Georgia | -9.020 | 4.867 | ▲153.96% | |
| Kazakhstan | -5.256 | 4.470 | ▲185.05% | |
| Kyrgyz Republic | -3.427 | 4.165 | ▲182.29% | |
| North Macedonia | -1.735 | 2.637 | ▲165.79% | |
| Russian Federation | -4.922 | 2.037 | ▲341.63% | |
| Tajikistan | -9.282 | 7.052 | ▲231.63% | |
| Turkmenistan | 1.171 | 8.720 | ▲86.57% | |
| Ukraine | -8.935 | 0.137 | ▲6612.15% | |
| Uzbekistan | -0.299 | 6.501 | ▲104.60% | |
| **Developing Countries** | | | | |
| **Country** | **1990-1999** | **2010-2019** | **Increase / Decrease in GDP** | |
| Algeria | 1.570 | 2.680 | ▲41.42% | |
| Argentina | 3.990 | 1.382 | ▼188.70% | |
| Barbados | 0.470 | -0.120 | ▼492.85% | |
| Belize | 5.954 | 2.152 | ▼176.62% | |
| Bolivia | 3.994 | 4.647 | ▲14.06% | |
| Botswana | 5.399 | 4.721 | ▼14.35% | |
| Brazil | 1.755 | 1.444 | ▼21.55% | |
| Cabo Verde | 10.134 | 2.755 | ▼267.91% | |
| Cameroon | 0.180 | 4.279 | ▲95.79% | |
| Chile | 6.096 | 3.336 | ▼82.76% | |
| China | 9.995 | 7.678 | ▼30.18% | |
| Colombia | 2.858 | 3.714 | ▲23.04% | |
| Costa Rica | 4.899 | 3.773 | ▼29.83% | |
| Cote d'Ivoire | 2.925 | 6.409 | ▲54.35% | |
| Cuba | -1.996 | 2.085 | ▲195.72% | |
| Dominica | 2.344 | 0.561 | ▼318.04% | |
| Dominican Republic | 4.988 | 5.640 | ▲11.57% | |
| Ecuador | 2.316 | 2.831 | ▲18.21% | |
| Egypt, Arab Republic | 4.489 | 3.801 | ▼18.10% | |
| El Salvador | 3.736 | 2.469 | ▼51.31% | |
| Equatorial Guinea | 34.314 | -3.312 | ▼1136.13% | |
| Eswatini | 4.884 | 2.652 | ▼84.13% | |
| Fiji | 3.163 | 3.308 | ▲4.37% | |
| Gabon | 2.476 | 4.059 | ▲39.01% | |
| Ghana | 4.265 | 6.774 | ▲37.04% | |
| Grenada | 3.452 | 2.845 | ▼21.31% | |
| Guatemala | 4.067 | 3.520 | ▼15.54% | |
| Guyana | 4.790 | 3.786 | ▼26.52% | |
| Honduras | 2.777 | 3.662 | ▲24.18% | |
| India | 5.769 | 6.623 | ▲12.90% | |
| Indonesia | 4.294 | 5.416 | ▲20.72% | |
| Iran Islamic Republic | 4.062 | 1.471 | ▼176.10% | |
| Iraq | 14.733 | 6.055 | ▼143.32% | |
| Jamaica | 2.160 | 0.694 | ▼211.15% | |
| Jordan | 4.314 | 2.394 | ▼80.18% | |
| Kenya | 2.244 | 5.035 | ▲55.43% | |
| Korea Republic | 7.301 | 3.333 | ▼119.08% | |
| Lebanon | 13.322 | 1.184 | ▼1025.22% | |
| Malaysia | 7.248 | 5.353 | ▼35.40% | |
| Marshall Islands | 1.118 | 2.323 | ▲51.87% | |
| Mauritius | 5.160 | 3.703 | ▼39.33% | |
| Mexico | 3.506 | 2.667 | ▼31.44% | |
| Micronesia Federal States | 2.478 | 0.719 | ▼244.80% | |
| Mongolia | -0.325 | 7.836 | ▲104.15% | |
| Morocco | 3.210 | 3.488 | ▲7.97% | |
| Namibia | 3.552 | 3.131 | ▼13.47% | |
| Nicaragua | 3.006 | 3.378 | ▲11.01% | |
| Pakistan | 3.976 | 4.027 | ▲1.29% | |
| Panama | 5.758 | 6.183 | ▲6.88% | |
| Paraguay | 3.091 | 4.311 | ▲28.30% | |
| Peru | 3.204 | 4.497 | ▲28.75% | |
| Philippines | 2.812 | 6.408 | ▲56.11% | |
| Samoa | 1.351 | 1.888 | ▲28.44% | |
| Saudi Arabia | 3.647 | 3.468 | ▼5.14% | |
| Seychelles | 4.864 | 4.741 | ▼2.60% | |
| Singapore | 7.219 | 4.988 | ▼44.74% | |
| South Africa | 1.386 | 1.725 | ▲19.64% | |
| Sri Lanka | 5.260 | 5.260 | ▼0.02% | |
| St. Kitts and Nevis | 4.017 | 2.747 | ▼46.20% | |
| St. Lucia | 3.333 | 1.412 | ▼136.05% | |
| St. Vincent and the Grenadines | 3.403 | 1.176 | ▼189.31% | |
| Syrian Arab Republic | 6.285 | -6.665 | ▼194.30% | |
| Thailand | 5.201 | 3.639 | ▼42.94% | |
| Tonga | 2.989 | 2.288 | ▼30.67% | |
| Trinidad and Tobago | 5.223 | -0.231 | ▼2364.34% | |
| Tunisia | 5.081 | 1.893 | ▼168.36% | |
| Turkey | 3.998 | 5.862 | ▲31.80% | |
| United Arab Emirates | 5.456 | 3.750 | ▼45.51% | |
| Uruguay | 3.696 | 2.890 | ▼27.92% | |
| Vanuatu | 3.700 | 2.652 | ▼39.51% | |
| Vietnam | 7.419 | 6.527 | ▼13.66% | |
| Zimbabwe | 2.906 | 6.083 | ▲52.23% | |
