# Supplementary material for: The interconnectedness of energy consumption with economic growth: A granger causality analysis
Source: Heliyon. 2024 Aug 28;10(17):e36709. doi: 10.1016/j.heliyon.2024.e36709 (PMC11402754; doi:10.1016/j.heliyon.2024.e36709)
Supplement: Multimedia component 5 [file mmc5.docx]

**Appendix E. Descriptive Statistics of Country Categories**

|  |  | **REC** | **NREC** | **GDP** |
| --- | --- | --- | --- | --- |
| Least-developed Countries | Observations | 1,140 | 1,140 | 1,140 |
|  | Mean | 73.3135 | 26.6864 | 4.0291 |
|  | Standard Deviation | 21.7491 | 21.7491 | 5.4025 |
|  | Minimum | 0.7000 | 1.6600 | -50.2480 |
|  | Maximum | 98.3400 | 99.3000 | 35.2240 |
| Developed Countries | Observations | 870 | 870 | 870 |
|  | Mean | 14.6202 | 85.3797 | 2.2892 |
|  | Standard Deviation | 13.4035 | 13.4035 | 2.9361 |
|  | Minimum | 0.3348 | 37.6300 | -14.1153 |
|  | Maximum | 62.3700 | 99.6651 | 25.17624 |
| Transitional economies | Observations | 390 | 390 | 390 |
|  | Mean | 15.1534 | 84.8465 | 2.2931 |
|  | Standard Deviation | 17.9644 | 17.9644 | 8.9556 |
|  | Minimum | 0.0017 | 35.4200 | -44.9000 |
|  | Maximum | 64.5800 | 99.9982 | 35.3845 |
| Developing Countries | Observations | 2,160 | 2,160 | 2,160 |
|  | Mean | 28.0851 | 71.9148 | 3.9831 |
|  | Standard Deviation | 24.4948 | 24.4948 | 6.2456 |
|  | Minimum | 0.00903 | 7.7369 | -64.0471 |
|  | Maximum | 92.2630 | 99.9909 | 149.9730 |
| Global view | Observations | 4,560 | 4,560 | 4,560 |
|  | Mean | 35.7172 | 64.2827 | 3.5269 |
|  | Standard Deviation | 31.08463 | 31.0846 | 5.9021 |
|  | Minimum | 0.0017 | 1.6600 | -64.0471 |
