# Supplementary material for: The interconnectedness of energy consumption with economic growth: A granger causality analysis
Source: Heliyon. 2024 Aug 28;10(17):e36709. doi: 10.1016/j.heliyon.2024.e36709 (PMC11402754; doi:10.1016/j.heliyon.2024.e36709)
Supplement: Multimedia component 6 [file mmc6.docx]

**Appendix F. Cross-country Analysis from Descriptive Statistics**

| **Least-developed Countries** | **REC (% of total final energy consumption)**  **)** | | | |  | | | **NREC (100 - % of total final energy consumption)** | | | | | | | |  | **GDP (% Annual rate)** | | | | | | |  |
| --- | --- | --- | --- | --- | --- | --- | --- | --- | --- | --- | --- | --- | --- | --- | --- | --- | --- | --- | --- | --- | --- | --- | --- | --- |
|  | **Mean** | **SD** | **MIN** | **MAX** |  | | **Mean** | | | **SD** | | **MIN** | | **MAX** |  | | **Mean** | | **SD** | **MIN** | | | **MAX** |  |
| Angola | 62.855 | 10.15 | 47.84 | 76.82 |  | | 37.15 | | | 10.15 | | 23.18 | | 52.16 |  | | 4.07 | | 7.89 | -23.98 | | | 15.03 |  |
| Bangladesh | 50.07 | 14.69 | 24.75 | 73.16 |  | | 49.93 | | | 14.69 | | 26.84 | | 75.25 |  | | 5.62 | | 1.10 | 3.49 | | | 7.88 |  |
| Benin | 65.74 | 18.29 | 43.97 | 94.99 |  | | 34.26 | | | 18.29 | | 5.01 | | 56.03 |  | | 4.66 | | 1.79 | 1.71 | | | 8.98 |  |
| Bhutan | 90.67 | 3.95 | 82.22 | 95.92 |  | | 9.33 | | | 3.95 | | 4.08 | | 17.78 |  | | 6.51 | | 3.45 | -0.41 | | | 18.36 |  |
| Burkina Faso | 82.94 | 8.48 | 64.85 | 93.61 |  | | 17.06 | | | 8.48 | | 6.39 | | 35.15 |  | | 5.47 | | 2.59 | -0.60 | | | 11.01 |  |
| Burundi | 93.25 | 3.01 | 84.77 | 96.04 |  | | 6.75 | | | 3.01 | | 3.96 | | 15.23 |  | | 1.19 | | 3.98 | -8.00 | | | 5.41 |  |
| Central African Republic | 91.89 | 2.16 | 85.41 | 95.08 |  | | 8.11 | | | 2.16 | | 4.92 | | 14.59 |  | | 1.27 | | 8.00 | -36.39 | | | 8.59 |  |
| Chad | 86.93 | 8.61 | 76.45 | 98.30 |  | | 13.07 | | | 8.61 | | 1.69 | | 23.55 |  | | 5.09 | | 8.56 | -15.71 | | | 33.63 |  |
| Comoros | 61.45 | 7.62 | 49.59 | 74.36 |  | | 38.54 | | | 7.62 | | 25.64 | | 50.41 |  | | 2.66 | | 3.08 | -5.40 | | | 10.85 |  |
| Congo Demographic Republic | 96.48 | 1.44 | 92.05 | 98.34 |  | | 3.52 | | | 1.44 | | 1.66 | | 7.95 |  | | 1.35 | | 6.27 | -13.47 | | | 9.47 |  |
| Congo Republic | 66.63 | 6.44 | 54.80 | 80.15 |  | | 33.37 | | | 6.44 | | 19.85 | | 45.20 |  | | 2.00 | | 5.40 | -10.78 | | | 11.64 |  |
| Ethiopia | 94.22 | 2.39 | 88.92 | 97.74 |  | | 5.77 | | | 2.39 | | 2.26 | | 11.08 |  | | 6.85 | | 5.90 | -8.67 | | | 13.57 |  |
| Gambia | 57.40 | 4.71 | 4802 | 62.94 |  | | 42.59 | | | 4.71 | | 37.06 | | 51.98 |  | | 3.16 | | 3.57 | -8.13 | | | 7.23 |  |
| Guinea | 81.28 | 6.11 | 65.44 | 89.93 |  | | 18.72 | | | 6.11 | | 10.07 | | 34.56 |  | | 4.36 | | 2.35 | -1.12 | | | 10.82 |  |
| Guinea-Bissau | 88.32 | 1.22 | 86.24 | 91.24 |  | | 11.68 | | | 1.22 | | 8.76 | | 13.76 |  | | 2.62 | | 6.41 | -28.10 | | | 11.60 |  |
| Haiti | 80.28 | 3.57 | 76.12 | 95.04 |  | | 19.72 | | | 3.57 | | 4.96 | | 23.88 |  | | 1.22 | | 4.11 | -11.95 | | | 9.90 |  |
| Kiribati | 32.12 | 20.21 | 3.75 | 55.85 |  | | 67.88 | | | 20.21 | | 44.15 | | 96.25 |  | | 1.67 | | 2.92 | -2.09 | | | 9.87 |  |
| Lao PDR | 72.50 | 13.58 | 48.43 | 88.45 |  | | 27.49 | | | 13.58 | | 11.56 | | 51.57 |  | | 6.81 | | 1.15 | 3.97 | | | 8.62 |  |
| Lesotho | 50.14 | 5.86 | 39.47 | 56.88 |  | | 49.86 | | | 5.86 | | 43.12 | | 60.53 |  | | 3.30 | | 2.49 | -3.14 | | | 6.97 |  |
| Madagascar | 83.45 | 2.82 | 76.29 | 87.35 |  | | 16.55 | | | 2.82 | | 12.65 | | 23.71 |  | | 2.58 | | 4.12 | -12.41 | | | 9.78 |  |
| Malawi | 80.93 | 2.69 | 73.02 | 84.03 |  | | 19.07 | | | 2.69 | | 15.96 | | 26.98 |  | | 4.26 | | 5.07 | -10.24 | | | 16.73 |  |
| Mali | 82.41 | 4.39 | 74.99 | 88.64 |  | | 17.59 | | | 4.39 | | 11.36 | | 25.01 |  | | 4.54 | | 3.80 | -3.22 | | | 15.38 |  |
| Mauritania | 38.27 | 7.06 | 24.70 | 46.99 |  | | 61.73 | | | 7.06 | | 53.00 | | 75.30 |  | | 3.29 | | 4.56 | -4.04 | | | 18.33 |  |
| Mozambique | 88.79 | 5.67 | 78.08 | 94.29 |  | | 11.21 | | | 5.67 | | 5.70 | | 21.92 |  | | 6.47 | | 3.88 | -6.12 | | | 12.09 |  |
| Myanmar | 80.08 | 8.94 | 57.85 | 91.12 |  | | 19.92 | | | 8.94 | | 8.88 | | 42.15 |  | | 8.48 | | 3.42 | 1.06 | | | 13.70 |  |
| Nepal | 88.02 | 5.29 | 74.27 | 95.12 |  | | 11.98 | | | 5.29 | | 4.88 | | 25.73 |  | | 4.64 | | 1.91 | 0.12 | | | 8.98 |  |
| Nigeria | 85.06 | 2.28 | 80.64 | 88.68 |  | | 14.94 | | | 2.28 | | 11.32 | | 19.36 |  | | 4.55 | | 3.99 | -2.04 | | | 15.33 |  |
| Papua New Guinea | 61.68 | 6.80 | 52.96 | 71.71 |  | 38.32 | | | 6.80 | | 28.29 | | 47.04 | | |  | 4.15 | 5.48 | | | -3.90 | 18.20 | | |
| Rwanda | 86.89 | 3.87 | 77.86 | 91.12 |  | 13.10 | | | 3.87 | | 8.88 | | 22.14 | | |  | 5.75 | 12.67 | | | -50.25 | 35.22 | | |
| Senegal | 45.98 | 6.25 | 36.15 | 55.55 |  | 54.02 | | | 6.25 | | 44.45 | | 63.85 | | |  | 3.67 | 2.16 | | | -0.75 | 7.39 | | |
| Sierra Leone | 84.89 | 6.34 | 73.6 | 91.37 |  | 15.10 | | | 6.34 | | 8.63 | | 26.40 | | |  | 3.08 | 9.34 | | | -20.60 | 26.42 | | |
| Solomon Islands | 52.56 | 6.90 | 41.83 | 66.76 |  | 47.44 | | | 6.90 | | 33.24 | | 58.17 | | |  | 3.26 | 5.22 | | | -14.28 | 12.70 | | |
| Sudan | 71.63 | 8.44 | 59.52 | 85.63 |  | 28.37 | | | 8.44 | | 14.37 | | 40.48 | | |  | 3.09 | 5.82 | | | -17.00 | 18.31 | | |
| Tanzania | 90.61 | 3.65 | 84.62 | 95.18 |  | 9.39 | | | 3.65 | | 4.82 | | 15.38 | | |  | 5.31 | 1.97 | | | 0.58 | 7.67 | | |
| Togo | 77.53 | 5.52 | 63.38 | 91.49 |  | 22.47 | | | 5.52 | | 8.50 | | 36.62 | | |  | 3.31 | 5.78 | | | -15.10 | 14.98 | | |
| Uganda | 93.91 | 1.94 | 90.07 | 96.84 |  | 6.09 | | | 1.94 | | 3.16 | | 9.93 | | |  | 6.45 | 2.20 | | | 3.13 | 11.52 | | |
| Yemen, Republic | 1.40 | 0.78 | 0.70 | 3.60 |  | 98.59 | | | 0.78 | | 96.40 | | 99.30 | | |  | 1.99 | 7.30 | | | -27.99 | 8.21 | | |
| Zambia | 86.64 | 2.89 | 81.89 | 90.32 |  | 13.36 | | | 2.89 | | 9.68 | | 18.11 | | |  | 4.34 | 3.85 | | | -8.63 | 10.30 | | |
| **Developed Countries** | **REC (% of total final energy consumption)** | | | |  | **NREC (100 - % of total final energy consumption)** | | | | | | | | | |  | **GDP (% Annual rate)** | | | | | | |  |
|  | **Mean** | **SD** | **MIN** | **MAX** |  | **Mean** | | | **SD** | | **MIN** | | **MAX** | | |  | **Mean** | **SD** | | | **MIN** | **MAX** | | |
| Andorra | 16.53 | 2.00 | 13.91 | 19.45 |  | 83.46 | | | 2.00 | | 80.55 | | 86.08 | | |  | 2.24 | 3.89 | | | -5.56 | 9.07 | | |
| Australia | 8.30 | 0.97 | 6.68 | 10.13 |  | 91.69 | | | 0.97 | | 89.87 | | 93.32 | | |  | 3.04 | 1.17 | | | -0.39 | 4.94 | | |
| Austria | 28.51 | 4.29 | 22.56 | 35.67 |  | 71.48 | | | 4.29 | | 64.33 | | 77.44 | | |  | 1.98 | 1.53 | | | -3.76 | 4.35 | | |
| Belgium | 4.16 | 3.47 | 0.93 | 10.66 |  | 95.83 | | | 3.47 | | 89.34 | | 99.06 | | |  | 1.87 | 1.34 | | | -2.02 | 3.79 | | |
| Bulgaria | 9.92 | 5.86 | 1.91 | 19.6 |  | 90.07 | | | 5.86 | | 80.4 | | 98.08 | | |  | 1.16 | 5.51 | | | -14.16 | 7.06 | | |
| Cyprus | 5.19 | 3.73 | 0.33 | 12.11 |  | 94.80 | | | 3.73 | | 87.89 | | 99.66 | | |  | 3.41 | 3.56 | | | -6.55 | 9.39 | | |
| Denmark | 17.56 | 10.12 | 7.04 | 37.52 |  | 82.43 | | | 10.12 | | 62.48 | | 92.95 | | |  | 1.76 | 1.80 | | | -4.97 | 5.33 | | |
| Finland | 32.90 | 6.53 | 24.02 | 45.76 |  | 67.09 | | | 6.53 | | 54.24 | | 75.97 | | |  | 1.72 | 3.29 | | | -8.07 | 6.33 | | |
| France | 11.10 | 1.99 | 8.52 | 15.53 |  | 88.89 | | | 1.99 | | 84.47 | | 91.48 | | |  | 1.63 | 1.33 | | | -2.87 | 3.92 | | |
| Germany | 7.93 | 5.27 | 1.98 | 17.17 |  | 92.06 | | | 5.27 | | 82.83 | | 98.01 | | |  | 1.64 | 2.06 | | | -5.69 | 5.26 | | |
| Greece | 10.53 | 3.96 | 7.11 | 18.51 |  | 89.46 | | | 3.96 | | 81.49 | | 92.89 | | |  | 0.96 | 3.78 | | | -10.15 | 5.79 | | |
| Hungary | 9.00 | 4.69 | 3.85 | 17.18 |  | 90.99 | | | 4.69 | | 82.82 | | 96.14 | | |  | 2.15 | 2.69 | | | -6.59 | 5.35 | | |
| Ireland | 4.54 | 3.20 | 1.90 | 12.34 |  | 95.45 | | | 3.20 | | 87.66 | | 98.09 | | |  | 5.64 | 5.49 | | | -5.10 | 25.18 | | |
| Italy | 9.25 | 5.02 | 3.78 | 17.27 |  | 90.74 | | | 5.02 | | 82.73 | | 96.21 | | |  | 0.77 | 1.77 | | | -5.28 | 3.79 | | |
| Japan | 4.71 | 1.10 | 3.50 | 7.69 |  | 95.28 | | | 1.10 | | 92.31 | | 96.50 | | |  | 1.06 | 1.94 | | | -5.69 | 4.84 | | |
| Luxembourg | 5.27 | 4.46 | 1.28 | 16.45 |  | 94.72 | | | 4.46 | | 83.55 | | 98.72 | | |  | 3.54 | 2.63 | | | -3.24 | 8.64 | | |
| Netherlands | 3.27 | 2.09 | 1.16 | 8.54 |  | 96.72 | | | 2.09 | | 91.46 | | 98.83 | | |  | 2.15 | 1.82 | | | -3.67 | 5.03 | | |
| New Zealand | 29.82 | 1.29 | 27.13 | 32.65 |  | 70.17 | | | 1.29 | | 67.35 | | 72.87 | | |  | 2.84 | 1.84 | | | -1.11 | 6.39 | | |
| North America | 8.13 | 1.70 | 6.06 | 10.68 |  | 91.86 | | | 1.70 | | 89.31 | | 93.93 | | |  | 2.45 | 1.52 | | | -2.63 | 4.74 | | |
| Norway | 58.86 | 1.70 | 56.23 | 62.37 |  | 41.13 | | | 1.70 | | 37.63 | | 43.77 | | |  | 2.29 | 1.53 | | | -1.73 | 5.28 | | |
| Poland | 7.89 | 2.82 | 2.06 | 12.18 |  | 92.10 | | | 2.82 | | 87.82 | | 97.93 | | |  | 3.79 | 2.59 | | | -7.02 | 7.10 | | |
| Portugal | 24.32 | 3.38 | 18.07 | 30.46 |  | 75.67 | | | 3.38 | | 69.54 | | 81.93 | | |  | 1.58 | 2.36 | | | -4.06 | 4.81 | | |
| Romania | 16.82 | 6.44 | 3.35 | 24.40 |  | 83.17 | | | 6.44 | | 75.60 | | 96.64 | | |  | 2.19 | 5.29 | | | -12.92 | 10.43 | | |
| Slovak Republic | 7.44 | 4.06 | 2.08 | 17.64 |  | 92.55 | | | 4.06 | | 82.36 82.36 | | 97.91 | | |  | 4.06 | 2.98 | | | -5.46 | 10.83 | | |
| Spain | 11.41 | 3.71 | 7.30 | 17.42 |  | 88.58 | | | 3.71 | | 82.58 | | 92.70 | | |  | 2.12 | 2.23 | | | -3.77 | 5.25 | | |
| Sweden | 40.77 | 7.11 | 31.35 | 52.88 |  | 59.22 | | | 7.11 | | 47.12 | | 68.64 | | |  | 2.14 | 2.33 | | | -4.34 | 5.95 | | |
| Switzerland | 19.80 | 2.50 | 17.11 | 24.99 |  | 80.19 | | | 2.50 | | 75.01 | | 82.88 | | |  | 1.73 | 1.52 | | | -2.08 | 4.02 | | |
| United Kingdom | 3.36 | 3.51 | 0.60 | 12.24 |  | 96.63 | | | 3.51 | | 87.76 | | 99.39 | | |  | 1.96 | 1.66 | | | -4.25 | 4.91 | | |
| United States | 6.54 | 2.09 | 4.08 | 10.42 |  | 93.45 | | | 2.09 | | 89.58 | | 95.91 | | |  | 2.46 | 1.53 | | | -2.59 | 4.79 | | |
| **Transitional economies** | **REC (% of total final energy consumption)** | | | |  | **NREC (100 - % of total final energy consumption)** | | | | | | | | | |  | **GDP (% Annual rate)** | | | | | | |  |
|  | **Mean** | **SD** | **MIN** | **MAX** |  | **Mean** | | | **SD** | | **MIN** | | **MAX** | | |  | **Mean** | **SD** | | | **MIN** | **MAX** | | |
| Albania | 40.12 | 7.10 | 25.522 | 55.95 |  | 59.88 | | | 7.10 | | 44.05 | | 74.48 | | |  | 3.04 | 8.05 | | | -28.00 | 13.32 | | |
| Armenia | 8.75 | 3.18 | 2.12 | 17.76 |  | 91.25 | | | 3.18 | | 82.24 | | 97.88 | | |  | 3.04 | 11.19 | | | -41.80 | 14.00 | | |
| Azerbaijan | 2.32 | 0.87 | 0.72 | 4.45 |  | 97.68 | | | 0.87 | | 95.55 | | 99.28 | | |  | 3.55 | 13.15 | | | -23.10 | 34.50 | | |
| Belarus | 5.37 | 2.37 | 0.82 | 7.83 |  | 94.63 | | | 2.37 | | 92.17 | | 99.18 | | |  | 2.27 | 6.67 | | | -11.70 | 11.45 | | |
| Georgia | 36.02 | 12.081 | 12.77 | 56.76 |  | 63.98 | | | 12.08 | | 43.24 | | 87.23 | | |  | 0.59 | 12.76 | | | -44.90 | 12.58 | | |
| Kazakhstan | 1.72 | 0.42 | 1.15 | 2.77 |  | 98.28 | | | 0.42 | | 97.23 | | 98.85 | | |  | 2.60 | 7.07 | | | -12.60 | 13.50 | | |
| Kyrgyz Republic | 25.22 | 6.77 | 7.93 | 36.00 |  | 74.78 | | | 6.77 | | 64.00 | | 92.07 | | |  | 1.83 | 7.44 | | | -20.09 | 10.92 | | |
| North Macedonia | 17.04 | 4.46 | 2.41 | 23.91 |  | 82.96 | | | 4.46 | | 76.09 | | 97.59 | | |  | 1.34 | 3.75 | | | -7.47 | 6.47 | | |
| Russian Federation | 3.54 | 0.25 | 3.18 | 4.04 |  | 96.46 | | | 0.25 | | 95.96 | | 96.82 | | |  | 0.87 | 6.32 | | | -14.53 | 10.00 | | |
| Tajikistan | 53.11 | 10.59 | 29.64 | 64.58 |  | 46.89 | | | 10.59 | | 35.42 | | 70.36 | | |  | 2.03 | 10.51 | | | -29.00 | 11.00 | | |
| Turkmenistan | 0 .07 | 0.07 | 0.00 | 0.32 |  | 99.93 | | | 0.07 | | 99.68 | | 99.998 | | |  | 5.77 | 10.06 | | | -17.30 | 35.38 | | |
| Ukraine | 2.42 | 1.94 | 0.60 | 7.44 |  | 97.58 | | | 1.94 | | 92.56 | | 99.40 | | |  | -1.36 | 8.64 | | | -22.90 | 11.80 | | |
| Uzbekistan | 1.29 | 0.30 | 0.72 | 1.75 |  | 98.70 | | | 0.30 | | 98.25 | | 99.28 | | |  | 4.22 | 4.50 | | | -11.20 | 9.47 | | |
| **Developing Countries** | **REC (% of total final energy consumption)** | | | |  | **NREC (100 - % of total final energy consumption)** | | | | | | | | | |  | **GDP (% Annual rate)** | | | | | | |  |
|  | **Mean** | **SD** | **MIN** | **MAX** |  | **Mean** | | | **SD** | | **MIN** | | **MAX** | | |  | **Mean** | **SD** | | | **MIN** | **MAX** | | |
| Algeria | 0.32 | 0.16 | 0.06 | 0.58 |  | 99.66 | | | 0.16 | | 99.42 | | 99.94 | | |  | 2.71 | 2.07 | | | -2.10 | 7.20 | | |
| Argentina | 9.78 | 1.08 | 7.65 | 11.62 |  | 90.22 | | | 1.08 | | 88.38 | | 92.35 | | |  | 2.65 | 5.66 | | | -10.89 | 10.13 | | |
| Barbados | 10.37 | 5.00 | 3.16 | 18.94 |  | 89.63 | | | 5.00 | | 81.06 | | 96.84 | | |  | 0.59 | 2.92 | | | -5.69 | 6.11 | | |
| Belize | 35.05 | 4.69 | 29.09 | 50.07 |  | 64.95 | | | 4.69 | | 49.93 | | 70.91 | | |  | 4.31 | 3.82 | | | -0.18 | 13.02 | | |
| Bolivia | 22.99 | 10.74 | 7.27 | 38.28 |  | 77.01 | | | 10.74 | | 61.72 | | 92.73 | | |  | 4.11 | 1.41 | | | 0.43 | 6.80 | | |
| Botswana | 34.87 | 8.22 | 25.38 | 48.27 |  | 65.13 | | | 8.22 | | 51.73 | | 74.62 | | |  | 4.55 | 4.06 | | | -7.65 | 11.34 | | |
| Brazil | 45.06 | 2.33 | 41.33 | 49.86 |  | 54.39 | | | 2.33 | | 50.14 | | 58.67 | | |  | 2.20 | 2.86 | | | -4.35 | 7.53 | | |
| Cabo Verde | 28.39 | 6.62 | 20.78 | 40.41 |  | 71.61 | | | 6.62 | | 59.59 | | 79.22 | | |  | 6.68 | 5.31 | | | -1.27 | 19.18 | | |
| Cameroon | 82.16 | 3.09 | 76.79 | 86.31 |  | 17.84 | | | 3.09 | | 13.69 | | 23.21 | | |  | 2.85 | 3.48 | | | -7.93 | 7.05 | | |
| Chile | 30.70 | 3.70 | 24.11 | 38.62 |  | 69.29 | | | 3.70 | | 61.38 | | 75.89 | | |  | 4.56 | 2.73 | | | -1.12 | 11.17 | | |
| China | 21.45 | 8.67 | 11.34 | 34.08 |  | 78.55 | | | 8.66 | | 65.91 | | 88.66 | | |  | 9.34 | 2.52 | | | 3.92 | 14.23 | | |
| Colombia | 30.89 | 2.83 | 25.80 | 38.25 |  | 69.11 | | | 2.83 | | 61.75 | | 74.19 | | |  | 3.49 | 2.24 | | | -4.20 | 6.95 | | |
| Costa Rica | 37.09 | 3.99 | 28.89 | 45.38 |  | 62.91 | | | 3.99 | | 54.62 | | 71.11 | | |  | 4.32 | 2.04 | | | -0.87 | 9.20 | | |
| Cote d'Ivoire | 71.19 | 5.36 | 61.93 | 79.11 |  | 28.81 | | | 5.36 | | 20.89 | | 38.07 | | |  | 3.33 | 4.41 | | | -5.37 | 10.76 | | |
| Cuba | 30.03 | 10.69 | 15.61 | 51.31 |  | 69.97 | | | 10.69 | | 48.68 | | 84.39 | | |  | 1.90 | 5.82 | | | -14.88 | 12.07 | | |
| Dominica | 11.93 | 2.92 | 8.05 20.848 | 20.85 |  | 88.07 | | | 2.92 | | 79.15 | | 91.95 | | |  | 1.85 | 3.11 | | | -6.62 | 7.12 | | |
| Dominican Republic | 19.33 | 3.77 | 13.98 | 28.16 |  | 80.67 | | | 3.77 | | 71.84 | | 86.02 | | |  | 4.98 | 3.46 | | | -5.45 | 11.22 | | |
| Ecuador | 16.98 | 3.48 | 11.79 | 24.19 |  | 83.02 | | | 3.48 | | 75.80 | | 88.21 | | |  | 3.01 | 2.64 | | | -4.74 | 8.21 | | |
| Egypt, Arab Republic | 7.04 | 1.50 | 5.10 | 9.83 |  | 92.96 | | | 1.50 | | 90.17 | | 94.90 | | |  | 4.42 | 1.59 | | | 1.13 | 7.16 | | |
| El Salvador | 38.64 | 14.67 | 19.39 | 67.17 |  | 61.34 | | | 14.67 | | 32.86 | | 80.61 | | |  | 2.57 | 1.74 | | | -2.09 | 7.02 | | |
| Equatorial Guinea | 34.07 | 33.62 | 3.54 | 84.71 |  | 65.93 | | | 33.62 | | 15.29 | | 96.46 | | |  | 17.40 | 31.47 | | | -9.11 | 149.97 | | |
| Eswatini | 69.61 | 10.61 | 47.93 | 92.26 |  | 30.39 | | | 10.61 | | 7.74 | | 52.07 | | |  | 3.63 | 3.58 | | | 0.82 | 21.02 | | |
| Fiji | 42.79 | 12.24 | 25.80 | 63.78 |  | 57.20 | | | 12.24 | | 36.22 | | 74.20 | | |  | 2.53 | 2.79 | | | -2.70 | 8.80 | | |
| Gabon | 79.84 | 6.75 | 69.67 | 90.13 |  | 20.16 | | | 6.75 | | 9.87 | | 30.33 | | |  | 2.37 | 3.66 | | | -8.93 | 7.09 | | |
| Ghana | 62.21 | 14.40 | 41.48 | 82.93 |  | 37.79 | | | 14.40 | | 17.07 | | 58.52 | | |  | 5.47 | 2.42 | | | 2.12 | 14.05 | | |
| Grenada | 9.89 | 1.44 | 6.15 | 11.48 |  | 90.11 | | | 1.44 | | 88.52 | | 93.85 | | |  | 2.93 | 4.41 | | | -6.61 | 13.28 | | |
| Guatemala | 66.03 | 3.28 | 61.35 | 74.97 |  | 33.97 | | | 3.28 | | 25.03 | | 38.65 | | |  | 3.67 | 1.08 | | | 0.48 | 6.34 | | |
| Guyana | 31.79 | 7.51 | 11.35 | 44.43 |  | 68.20 | | | 7.51 | | 55.57 | | 88.65 | | |  | 3.49 | 3.19 | | | -3.07 | 8.53 | | |
| Honduras | 55.37 | 7.45 | 45.92 | 70.20 |  | 44.63 | | | 7.45 | | 29.79 | | 54.08 | | |  | 3.65 | 2.55 | | | -3.32 | 7.29 | | |
| India | 44.21 | 9.12 | 32.41 | 58.65 |  | 55.79 | | | 9.12 | | 41.35 | | 67.59 | | |  | 6.23 | 1.92 | | | 1.06 | 8.85 | | |
| Indonesia | 40.49 | 11.04 | 19.09 | 58.59 |  | 59.51 | | | 11.04 | | 41.40 | | 80.91 | | |  | 4.94 | 3.68 | | | -13.13 | 8.22 | | |
| Iran Islamic Republic | 0.97 | 0.27 | 0.44 | 1.53 |  | 99.02 | | | 0.27 | | 98.47 | | 99.56 | | |  | 3.41 | 4.35 | | | -3.75 | 13.59 | | |
| Iraq | 0.88 | 0.65 | 0.31 | 2.56 |  | 99.12 | | | 0.65 | | 97.44 | | 99.69 | | |  | 8.53 | 22.46 | | | -64.05 | 57.82 | | |
| Jamaica | 8.97 | 2.03 | 4.82 | 12.16 |  | 91.03 | | | 2.03 | | 87.84 | | 95.18 | | |  | 1.26 | 2.41 | | | -4.35 | 9.42 | | |
| Jordan | 2.98 | 1.49 | 1.69 | 8.17 |  | 97.02 | | | 1.49 | | 91.83 | | 98.31 | | |  | 4.39 | 2.96 | | | -0.28 | 14.35 | | |
| Kenya | 77.95 | 3.64 | 68.08 | 83.25 |  | 22.05 | | | 3.64 | | 16.75 | | 31.92 | | |  | 3.62 | 2.19 | | | -0.80 | 8.06 | | |
| Korea, Republic | 1.35 | 0.88 | 0.44 | 3.36 |  | 98.65 | | | 0. .88 | | 96.64 | | 99.56 | | |  | 5.18 | 3.46 | | | -5.13 | 11.47 | | |
| Lebanon | 5.79 | 1.64 | 3.96 | 11.34 |  | 94.21 | | | 1.64 | | 88.66 | | 96.04 | | |  | 6.55 | 10.19 | | | -6.91 | 49.45 | | |
| Malaysia | 5.34 | 3.01 | 1.96 | 11.98 |  | 94.66 | | | 3.01 | | 88.02 | | 98.04 | | |  | 5.80 | 3.59 | | | -7.36 | 10.00 | | |
| Marshall Islands | 16.24 | 3.41 | 11.32 | 19.69 |  | 83.76 | | | 3.41 | | 80.31 | | 88.68 | | |  | 1.60 | 4.31 | | | -10.30 | 8.21 | | |
| Mauritius | 21.75 | 12.29 | 8.94 | 47.07 |  | 78.25 | | | 12.29 | | 52.93 | | 91.06 | | |  | 4.44 | 1.49 | | | 1.61 | 8.20 | | |
| Mexico | 10.95 | 1.67 | 8.97 | 14.41 |  | 89.05 | | | 1.67 | | 85.59 | | 91.03 | | |  | 2.55 | 2.93 | | | -6.29 | 6.85 | | |
| Micronesia Federal States | 1.43 | 0.21 | 0.86 | 1.95 |  | 98.57 | | | 0.21 | | 98.05 | | 99.14 | | |  | 1.23 | 3.40 | | | -6.06 | 8.14 | | |
| Mongolia | 3.92 | 1.24 | 1.89 | 6.51 |  | 96.08 | | | 1.24 | | 93.49 | | 98.11 | | |  | 4.51 | 5.82 | | | -9.26 | 17.29 | | |
| Morocco | 15.27 | 3.35 | 10.45 | 23.00 |  | 84.73 | | | 3.35 | | 77.00 | | 89.55 | | |  | 3.82 | 3.60 | | | -5.41 | 12.37 | | |
| Namibia | 33.62 | 3.44 | 28.99 | 43.18 |  | 66.38 | | | 3.44 | | 56.82 | | 71.01 | | |  | 3.69 | 2.99 | | | -1.58 | 12.27 | | |
| Nicaragua | 56.93 | 6.39 | 48.91 | 70.52 |  | 43.07 | | | 6.39 | | 29.48 | | 51.09 | | |  | 3.10 | 3.00 | | | -3.78 | 7.04 | | |
| Pakistan | 49.29 | 4.22 | 42.09 | 58.09 |  | 50.70 | | | 4.22 | | 41.91 | | 57.91 | | |  | 4.18 | 1.72 | | | 1.01 | 7.71 | | |
| Panama | 28.01 | 7.80 | 18.91 | 43.59 |  | 71.99 | | | 7.80 | | 56.41 | | 81.09 | | |  | 5.85 | 3.00 | | | 0.57 | 11.98 | | |
| Paraguay | 67.11 | 5.11 | 59.24 | 79.15 |  | 32.89 | | | 5.12 | | 20.85 | | 40.76 | | |  | 3.26 | 3.08 | | | -2.31 | 11.10 | | |
| Peru | 33.87 | 4.68 | 27.12 | 41.23 |  | 66.13 | | | 4.68 | | 58.77 | | 72.88 | | |  | 4.25 | 3.46 | | | -4.98 | 12.31 | | |
| Philippines | 34.94 | 6.73 | 26.73 | 51.96 |  | 65.06 | | | 6.73 | | 48.04 | | 73.27 | | |  | 4.59 | 2.23 | | | -0.51 | 7.33 | | |
| Samoa | 44.351 | 5.42 | 34.21 | 59.69 |  | 55.65 | | | 5.42 | | 40.31 | | 65.79 | | |  | 2.37 | 3.44 | | | -4.42 | 8.13 | | |
| Saudi Arabia | 0.01 | 0.01 | 001 | 0.03 |  | 99.98 | | | 0.01 | | 99.96 | | 99.99 | | |  | 3.54 | 4.72 | | | -3.76 | 15.19 | | |
| Seychelles | 1.59 | 0.95 | 0.71 | 4.25 |  | 98.41 | | | 0.95 | | 95.75 | | 99.29 | | |  | 3.78 | 4.37 | | | -5.89 | 11.96 | | |
| Singapore | 0.53 | 0.12 | 0.19 | 0.84 |  | 99.47 | | | 0.12 | | 99.16 | | 99.81 | | |  | 5.86 | 3.84 | | | -2.19 | 14.52 | | |
| South Africa | 13.62 | 3.35 | 9.77 | 18.59 |  | 86.36 | | | 3.35 | | 81.41 | | 90.23 | | |  | 2.24 | 2.00 | | | -2.14 | 5.60 | | |
| Sri Lanka | 62.56 | 7.59 | 49.33 | 78.09 |  | 37.44 | | | 7.59 | | 21.91 | | 50.67 | | |  | 5.17 | 2.06 | | | -1.55 | 9.14 | | |
| State Kitts and Nevis | 17.20 | 15.98 | 0.01 | 41.34 |  | 82.79 | | | 15.98 | | 58.66 | | 99.99 | | |  | 3.52 | 3.77 | | | -3.93 | 11.18 | | |
| State Lucia | 11.33 | 6.63 | 2.53 | 24.05 |  | 88.67 | | | 6.63 | | 75.95 | | 97.47 | | |  | 2.19 | 3.31 | | | -3.41 | 9.89 | | |
| State Vincent and the Grenadines | 7.01 | 2.99 | 3.32 | 15.44 |  | 92.99 | | | 2.99 | | 84.56 | | 96.68 | | |  | 2.57 | 2.69 | | | -4.47 | 7.77 | | |
| Syrian Arab Republic | 1.75 | 0.58 | 0.58 | 3.07 |  | 98.24 | | | 0.58 | | 96.93 | | 99.42 | | |  | 1.44 | 9.00 | | | -26.34 | 12.97 | | |
| Thailand | 23.27 | 3.58 | 19.89 | 33.64 |  | 76.73 | | | 3.58 | | 66.36 | | 80.11 | | |  | 4.39 | 3.80 | | | -7.63 | 11.17 | | |
| Tonga | 1.69 | 0.46 | 1.01 | 2.70 |  | 98.30 | | | 0.46 | | 97.3 | | 98.99 | | |  | 2.06 | 2.89 | | | -5.20 | 7.38 | | |
| Trinidad and Tobago | 0.70 | 0.34 | 0.35 | 1.35 |  | 99.29 | | | 0.34 | | 98.65 | | 99.65 | | |  | 3.82 | 4.84 | | | -6.32 | 14.44 | | |
| Tunisia | 13.79 | 0.99 | 11.82 | 16.07 |  | 86.20 | | | 0.99 | | 83.93 | | 88.18 | | |  | 3.77 | 2.24 | | | -2.05 | 7.95 | | |
| Turkey | 17.01 | 4.54 | 11.4 | 24.51 |  | 82.99 | | | 4.55 | | 75.49 | | 88.60 | | |  | 4.63 | 4.55 | | | -5.75 | 11.20 | | |
| United Arab Emirates | 0.14 | 0.11 | 0.06 | 0.67 |  | 99.86 | | | 0.11 | | 99.33 | | 99.94 | | |  | 4.70 | 4.24 | | | -5.24 | 18.33 | | |
| Uruguay | 46.09 | 8.19 | 33.29 | 60.86 |  | 53.91 | | | 8.19 | | 39.14 | | 66.70 | | |  | 2.92 | 3.88 | | | -7.73 | 8.55 | | |
| Vanuatu | 38.04 | 12.80 | 19.61 | 64.26 |  | 61.96 | | | 12.80 | | 35.74 | | 80.39 | | |  | 3.15 | 3.38 | | | -5.20 | 11.70 | | |
| Vietnam | 48.11 | 16.90 | 18.65 | 76.08 |  | 51.89 | | | 16.90 | | 23.92 | | 81.35 | | |  | 6.86 | 1.21 | | | 4.77 | 9.54 | | |
| Zimbabwe | 74.62 | 7.03 | 63.74 | 82.46 |  | 25.38 | | | 7.03 | | 17.54 | | 36.26 | | |  | 1.27 | 8.74 | | | -17.67 | 19.68 | | |
