# Supplementary material for: The interconnectedness of energy consumption with economic growth: A granger causality analysis
Source: Heliyon. 2024 Aug 28;10(17):e36709. doi: 10.1016/j.heliyon.2024.e36709 (PMC11402754; doi:10.1016/j.heliyon.2024.e36709)
Supplement: Multimedia component 7 [file mmc7.docx]

**Appendix G. Cross-country Analysis from Unit Root**

| **Least-developed Countries** | | | |
| --- | --- | --- | --- |
| **Country** | **DREC** | **DNREC** | **DGDP** |
| Angola | -2.1895** | -2.1895** | -4.3475* |
| Bangladesh | -1.7420** | -1.7420** | -2.0695** |
| Benin | -1.5915* | -1.5915* | -3.2042* |
| Bhutan | -1.7128* | -1.7128* | -3.4754* |
| Burkina Faso | -2.8827*** | -2.8827*** | -0.8809 |
| Burundi | -0.9523 | -0.9523 | -3.1343* |
| Central African Republic | -2.6795*** | -2.6795*** | -4.5388* |
| Chad | -3.1869*** | -3.1869*** | -4.6367* |
| Comoros | -3.3911*** | -3.3911*** | -6.1104* |
| Congo, Demographic Republic | -2.2942** | -2.2942** | -3.9649* |
| Congo Republic | -2.7554*** | -2.7554*** | -3.7647* |
| Ethiopia | -2.8883*** | -2.8883*** | -5.9464* |
| Gambia | -2.7561*** | -2.7561*** | -5.0155* |
| Guinea | -1.8699** | -1.8699** | -5.2185* |
| Guinea-Bissau | -1.6931** | -1.6931** | -5.1886* |
| Haiti | -3.1906*** | -3.1906*** | -2.8464* |
| Kiribati | -2.7751*** | -2.7751*** | -6.5770* |
| Lao PDR | -1.5168* | -1.5168* | -1.2635*** |
| Lesotho | -2.8611*** | -2.8611*** | -3.5034* |
| Madagascar | -1.8430** | -1.8430** | -3.7956* |
| Malawi | -1.3269* | -1.3269* | -3.1420* |
| Mali | -3.8247*** | -3.8247*** | -2.9351* |
| Mauritania | -5.0591*** | -5.0591*** | -4.9537* |
| Mozambique | -2.2990** | -2.2990** | -6.3432* |
| Myanmar | -1.3330* | -1.3330* | -2.3928* |
| Nepal | 0.4027 | 0.4027 | -6.2341* |
| Nigeria | -2.9246*** | -2.9246*** | -0.2051 |
| Papua New Guinea | -2.9889*** | -2.9889*** | -3.3342* |
| Rwanda | -0.0269 | -0.0269 | -4.9901* |
| Senegal | -2.8380*** | -2.8380*** | -2.1997* |
| Sierra Leone | -1.5049* | -1.5049* | -4.4246* |
| Solomon Islands | -1.7854** | -1.7854** | -3.5690* |
| Sudan | -1.4874* | -1.4874* | -4.3276* |
| Tanzania | -1.8682** | -1.8682** | -3.0892* |
| Togo | -2.5320*** | -2.5320*** | -3.7471* |
| Uganda | -1.9799** | -1.9799** | -4.1725* |
| Yemen Republic | -1.2119 | -1.2119 | -3.7799* |
| Zambia | -0.7829 | -0.7829 | -4.8770* |
| **Developed Countries** | | | |
| **Country** | **DREC** | **DNREC** | **DGDP** |
| Andorra | -1.1167 | -1.1167 | -2.9458* |
| Australia | -2.0762** | -2.0762** | -3.3421* |
| Austria | -0.7577 | -0.7577 | -4.7173* |
| Belgium | -0.0080 | -0.0080 | -5.5948* |
| Bulgaria | -2.9076* | -2.9076* | -2.0791* |
| Cyprus | -1.8067** | -1.8067** | -2.7105* |
| Denmark | -1.5061*** | -1.5061*** | -3.2840* |
| Finland | -2.1088** | -2.1088** | -4.2151* |
| France | -1.6164*** | -1.6164*** | -4.2043* |
| Germany | -1.3395*** | -1.3395*** | -5.4479* |
| Greece | -1.3864*** | -1.3864*** | -2.4444* |
| Hungary | -0.7109 | -0.7109 | -4.4437* |
| Ireland | 0.0138 | 0.0138 | -3.6290* |
| Italy | -2.3788* | -2.3788* | -6.3000* |
| Japan | -0.4121 | -0.4121 | -4.6586* |
| Luxembourg | -1.3784*** | -1.3784*** | -4.7903* |
| Netherlands | 0.8294 | 0.8294 | -4.0568* |
| New Zealand | -2.3155** | -2.3155** | -2.9439* |
| North America | -3.8380* | -3.8380* | -2.5991* |
| Norway | -2.6122* | -2.6122* | -4.0023* |
| Poland | -2.3036** | -2.3036** | -0.335 |
| Portugal | -4.5947* | -4.5947* | -4.4331* |
| Romania | -2.6995* | -2.6995* | -2.2900* |
| Slovak Republic | 0.6358 | 0.6358 | -4.4329* |
| Spain | -1.9408** | -1.9408** | -3.4934* |
| Sweden | -2.8577* | -2.8577* | -5.1840* |
| Switzerland | -1.6307*** | -1.6307*** | -2.8842* |
| United Kingdom | -0.1484 | -0.1484 | -4.0242* |
| United States | -4.2805* | -4.2805* | -2.3510* |
| **Transitional economies** | | | |
| **Country** | **DREC** | **DNREC** | **DGDP** |
| Albania | -4.3035* | -4.3035* | -3.17788* |
| Armenia | -2.6654* | -2.6654* | -5.1586* |
| Azerbaijan | -2.8403** | -2.8403** | -2.3495* |
| Belarus | -1.0289 | -1.0289 | -2.6957* |
| Georgia | -1.3144*** | -1.3144*** | -4.4197* |
| Kazakhstan | -2.5860** | -2.5860** | -5.0628* |
| Kyrgyz Republic | -1.7883** | -1.7883** | -2.6501* |
| North Macedonia | -4.5124* | -4.5124* | -2.7903* |
| Russian Federation | -4.1348* | -4.1348* | -3.3688* |
| Tajikistan | -1.4744*** | -1.4744*** | -4.4735* |
| Turkmenistan | -9.3944* | -9.3948* | -6.8484* |
| Ukraine | -1.9345** | -1.9345** | -3.6561* |
| Uzbekistan | -2.1089** | -2.1089** | -5.6040* |
| **Developing Countries** | | | |
| **Country** | **DREC** | **DNREC** | **DGDP** |
| Algeria | -1.0684 | -1.0684 | 3.9777* |
| Argentina | -3.8047*** | -3.8047*** | -3.8596* |
| Barbados | -4.2061*** | -4.2061*** | -2.8139* |
| Belize | -2.4039*** | -2.4039*** | -3.5330* |
| Bolivia | 1.4313 | 1.4313 | -2.9798* |
| Botswana | -2.2477 | -2.2477 | -5.3725* |
| Brazil | -1.6310* | -1.6310* | -3.1555* |
| Cabo Verde | -2.0041** | -2.0041** | -3.2149* |
| Cameroon | -2.1099** | -2.1099** | -3.1557* |
| Chile | -3.4050*** | -3.4050*** | -4.5937* |
| China | 0.0246 | 0.0246 | -4.3722* |
| Colombia | -4.5735*** | -4.5735*** | -3.9962* |
| Costa Rica | -4.0758*** | -4.0758*** | -5.0611* |
| Cote d'Ivoire | -1.6216* | -1.6216* | -2.3426* |
| Cuba | -2.3056** | -2.3056** | -1.6798** |
| Dominica | -2.9050*** | -2.9050*** | -2.4582* |
| Dominican Republic | -2.5959*** | -2.5959*** | -5.9619* |
| Ecuador | -3.6395*** | -3.6395*** | -5.3084* |
| Egypt Arab Republic | -3.2167*** | -3.2167*** | 0.4487 |
| El Salvador | -2.7050*** | -2.7050*** | -3.0698* |
| Equatorial Guinea | -0.4264 | -0.4264 | -4.2230* |
| Eswatini | -2.6216*** | -2.6216*** | 1.3930 |
| Fiji | -4.9564*** | -4.9564*** | -3.5773* |
| Gabon | -2.0022** | -2.0022** | -4.5422* |
| Ghana | -2.2192** | -2.2192** | -2.6182* |
| Grenada | -2.3199** | -2.3199** | -3.0632* |
| Guatemala | -1.6194* | -1.6194* | -5.0405* |
| Guyana | -1.4373* | -1.4373* | -3.0796* |
| Honduras | -1.4963* | -1.4963* | -4.9738* |
| India | -1.6280* | -1.6280* | -2.6207* |
| Indonesia | -2.1337** | -2.1337** | -4.0461* |
| Iran Islamic Republic | -2.7018*** | -2.7018*** | -4.2582* |
| Iraq | -2.9727*** | -2.9727*** | -1.7588** |
| Jamaica | -0.5286 | -0.5286 | -2.8840* |
| Jordan | 2.5259 | 2.5259 | -9.9956* |
| Kenya | -2.0551** | -2.0551** | -4.8672* |
| Korea Republic | -2.1302** | -2.1302** | -5.2742* |
| Lebanon | -0.9293 | -0.9293 | -1.4190** |
| Malaysia | -2.1316** | -2.1316** | -6.0374* |
| Marshall Islands | -1.0905 | -1.0905 | -4.3957* |
| Mauritius | -2.5777*** | -2.5777*** | -7.5996* |
| Mexico | -2.5175*** | -2.5175*** | -3.7432* |
| Micronesia Federal States | -4.0499*** | -4.0499*** | -3.0687* |
| Mongolia | -1.2160 | -1.2160 | -4.0343* |
| Morocco | -2.5588*** | -2.5588*** | -4.0343* |
| Namibia | -0.7471 | -0.7471 | -3.5617* |
| Nicaragua | -0.5668 | -0.5668 | -2.7959* |
| Pakistan | -2.4435*** | -2.4435*** | -2.6378* |
| Panama | -2.5664*** | -2.5664*** | -4.7923* |
| Paraguay | -2.3851*** | -2.3851*** | -5.3573* |
| Peru | -1.7510** | -1.7510** | -2.6239* |
| Philippines | -0.3931 | -0.3931 | -4.0502* |
| Samoa | -3.5956*** | -3.5956*** | -4.6604* |
| Saudi Arabia | -4.2947*** | -4.2947*** | -4.4374* |
| Seychelles | -1.5658* | -1.5658* | -3.9639* |
| Singapore | -0.9140 | -0.9140 | -6.6370* |
| South Africa | -1.0822 | -1.0822 | -3.5901* |
| Sri Lanka | -1.1687 | -1.1687 | -3.7970* |
| St. Kitts and Nevis | -2.3747*** | -2.3747*** | -5.2532* |
| St. Lucia | -2.1636** | -2.1636** | -2.3345* |
| St. Vincent and the Grenadines | -1.2173 | -1.2173 | -3.9298* |
| Syrian Arab Republic | -3.5743*** | -3.5743*** | -3.3712* |
| Thailand | -0.9972 | -0.9972 | -3.5085* |
| Tonga | -2.0213** | -2.0213** | -4.3893* |
| Trinidad and Tobago | 0.0159 | 0.0159 | -3.7993* |
| Tunisia | -3.3820*** | -3.3820*** | -2.3653* |
| Turkey | -2.9576*** | -2.9576*** | -3.4098* |
| United Arab Emirates | 11.8995 | 11.8995 | -2.5487* |
| Uruguay | -2.3960*** | -2.3960*** | -2.8568* |
| Vanuatu | -1.5070* | -1.5070* | -4.7074* |
| Vietnam | -2.6408*** | -2.6408*** | -4.0957* |
| Zimbabwe | -2.2307** | -2.2307** | -3.5638* |
