# Supplementary material for: The interconnectedness of energy consumption with economic growth: A granger causality analysis
Source: Heliyon. 2024 Aug 28;10(17):e36709. doi: 10.1016/j.heliyon.2024.e36709 (PMC11402754; doi:10.1016/j.heliyon.2024.e36709)
Supplement: Multimedia component 8 [file mmc8.docx]

**Appendix H. Graph of Eigenvalue Stability Condition**

|  | **REC- GDP** |  | **NREC- GDP** |
| --- | --- | --- | --- |
| Least-developed Countries | ***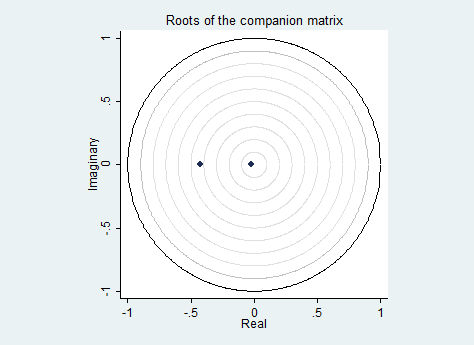*** |  | ***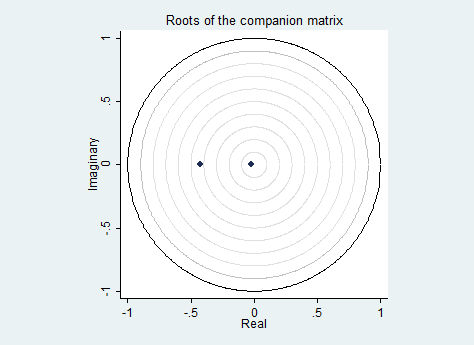*** |
| Developed Countries | ***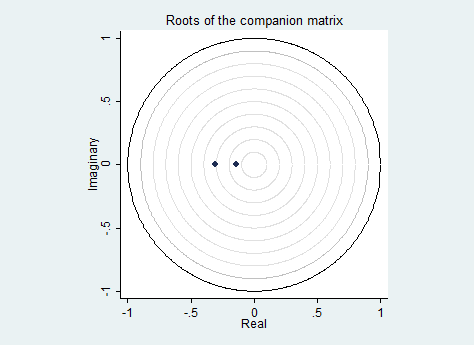*** |  | ***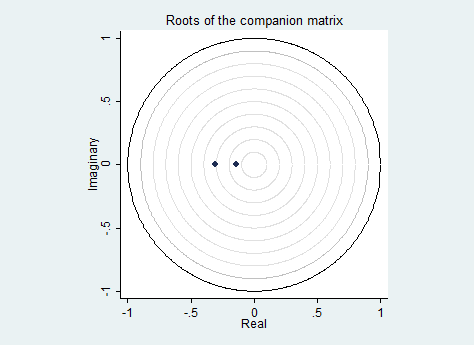*** |
| Transitional economies | ***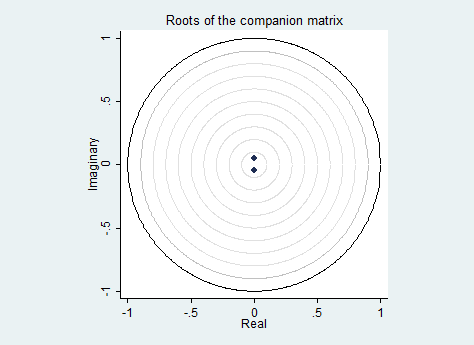*** |  | ***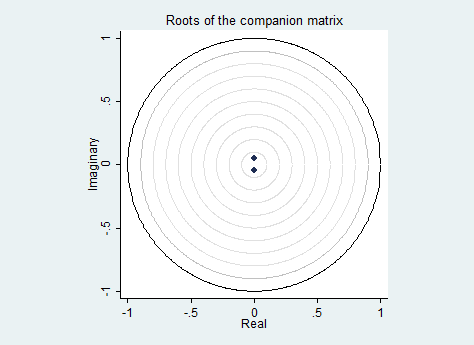*** |
| Developing Countries | ***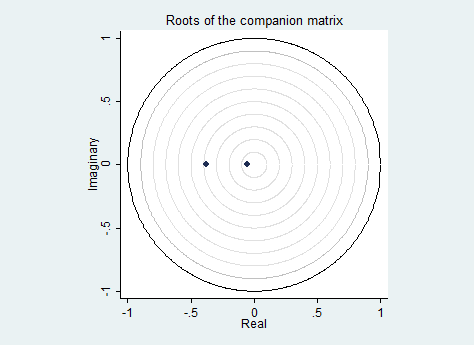*** |  | ***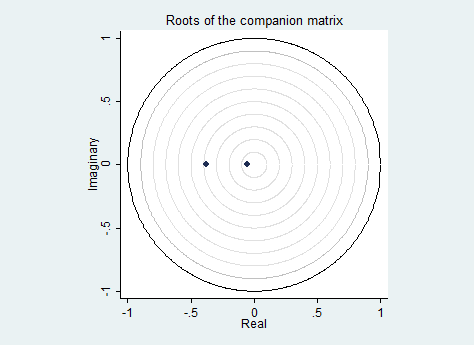*** |
