# Supplementary material for: The interconnectedness of energy consumption with economic growth: A granger causality analysis
Source: Heliyon. 2024 Aug 28;10(17):e36709. doi: 10.1016/j.heliyon.2024.e36709 (PMC11402754; doi:10.1016/j.heliyon.2024.e36709)
Supplement: Multimedia component 9 [file mmc9.docx]

**Appendix I. Cross-country Analysis from Granger causality between REC and GDP**

| **Least-developed Countries** | | | |
| --- | --- | --- | --- |
| **Country** | **DREC → DGDP** | **DGDP → DREC** | **DREC - DGDP** |
| Angola | 0.0029 | 0.4424 | **⇼** |
| Bangladesh | 2.7909*** | 1.2148 | ← |
| Benin | 0.0509 | 0.11437 | **⇼** |
| Bhutan | 0.2428 | 3.1147*** | → |
| Burkina Faso | 0.0701 | 1.1723 | **⇼** |
| Burundi | 0.0876 | 0.0032 | **⇼** |
| Central African Republic | 1.3538 | 1.5463 | **⇼** |
| Chad | 1.0285 | 4.0563** | → |
| Comoros | 0.3234 | 0.0153 | **⇼** |
| Congo, Demographic Republic | 0.0225 | 0.3737 | **⇼** |
| Congo Republic | 0.0054 | 1.8840 | **⇼** |
| Ethiopia | 0.2388 | 4.2060** | → |
| Gambia | 0.7478 | 0.6866 | **⇼** |
| Guinea | 0.2567 | 0.5733 | **⇼** |
| Guinea-Bissau | 13.3810* | 0.1972 | ← |
| Haiti | 0.0042 | 0.9744 | **⇼** |
| Kiribati | 2.0663 | 0.5507 | **⇼** |
| Lao PDR | 2.1315 | 0.1497 | **⇼** |
| Lesotho | 2.6560 | 0.0398 | **⇼** |
| Madagascar | 0.1951 | 0.0899 | **⇼** |
| Malawi | 0.4006 | 0.0508 | **⇼** |
| Mali | 0.0490 | 0.0512 | **⇼** |
| Mauritania | 1.76E+00 | 0.8101 | **⇼** |
| Mozambique | 0.3079 | 0.6983 | **⇼** |
| Myanmar | 2.5462 | 0.0191 | **⇼** |
| Nepal | 0.1237 | 3.5973** | → |
| Nigeria | 1.7349 | 0.0523 | **⇼** |
| Papua New Guinea | 0.5436 | 3.1939** | → |
| Rwanda | 4.8911** | 0.0509 | ← |
| Senegal | 1.5580 | 0.7263 | **⇼** |
| Sierra Leone | 0.3161 | 0.1339 | **⇼** |
| Solomon Islands | 0.0009 | 1.8244 | **⇼** |
| Sudan | 0.1854 | 0.0039 | **⇼** |
| Tanzania | 2.2014 | 0.1083 | **⇼** |
| Togo | 0.6593 | 0.6214 | **⇼** |
| Uganda | 8.889* | 0.0256 | ← |
| Yemen Republic | 1.5021 | 3.8678 | **⇼** |
| Zambia | 0.9805 | 0.1378 | **⇼** |
| **Developed Countries** | | | |
| **Country** | **DREC → DGDP** | **DGDP → DREC** | **DREC - DGDP** |
| Andorra | 0.1693 | 0.6997 | **⇼** |
| Australia | 0.1233 | 0.6806 | **⇼** |
| Austria | 0.8315 | 0.3933 | **⇼** |
| Belgium | 1.0817 | 0.1077 | **⇼** |
| Bulgaria | 5.9032** | 0.0386 | ← |
| Cyprus | 1.0412 | 6.8594* | → |
| Denmark | 1.3303 | 0.0196 | **⇼** |
| Finland | 2.4153 | 7.4684* | → |
| France | 3.4957*** | 0.0002 | ← |
| Germany | 0.9745 | 0.2554 | **⇼** |
| Greece | 4.0050** | 0.4542 | ← |
| Hungary | 0.2791 | 1.3633 | **⇼** |
| Ireland | 5.7045** | 1.4208 | ← |
| Italy | 6.7095** | 0.8962 | ← |
| Japan | 0.1913 | 0.0713 | **⇼** |
| Luxembourg | 2.1472 | 0.4289 | **⇼** |
| Netherlands | 15.3300* | 0.6866 | ← |
| New Zealand | 1.8157 | 0.0124 | **⇼** |
| North America | 0.2907 | 0.4985 | **⇼** |
| Norway | 3.5143*** | 12.8400* | **↔** |
| Poland | 7.1986* | 0.2888 | ← |
| Portugal | 1.6062 | 3.5690*** | → |
| Romania | 0.0396 | 1.2663 | **⇼** |
| Slovak Republic | 0.1484 | 5.5285** | → |
| Spain | 3.9688** | 1.6952 | ← |
| Sweden | 0.0202 | 0.2162 | **⇼** |
| Switzerland | 0.4010 | 0.4851 | **⇼** |
| United Kingdom | 0.9751 | 0.4423 | **⇼** |
| United States | 0.2784 | 0.6907 | **⇼** |
| **Transitional economies** | | | |
| **Country** | **DREC → DGDP** | **DGDP → DREC** | **DREC - DGDP** |
| Albania | 1.4267 | 10.0910* | → |
| Armenia | 1.7295 | 3.8877** | → |
| Azerbaijan | 0.0157 | 0.9696 | **⇼** |
| Belarus | 0.0579 | 1.5165 | **⇼** |
| Georgia | 0.1446 | 1.8844 | **⇼** |
| Kazakhstan | 0.3875 | 1.6366 | **⇼** |
| Kyrgyz Republic | 8.3908* | 1.1501 | ← |
| North Macedonia | 0.8105 | 0.0546 | **⇼** |
| Russian Federation | 4.1215** | 0.8126 | ← |
| Tajikistan | 0.2403 | 0.7460 | **⇼** |
| Turkmenistan | 12.1430* | 2.2027 | ← |
| Ukraine | 0.0220 | 0.2568 | **⇼** |
| Uzbekistan | 0.2609 | 0.1763 | **⇼** |
| **Developing Countries** | | | |
| **Country** | **DREC → DGDP** | **DGDP → DREC** | **DREC - DGDP** |
| Algeria | 0.8083 | 0.0225 | **⇼** |
| Argentina | 0.0395 | 0.2698 | **⇼** |
| Barbados | 0.3613 | 0.0566 | **⇼** |
| Belize | 1.3428 | 0.3455 | **⇼** |
| Bolivia | 0.2765 | 0.1074 | **⇼** |
| Botswana | 2.7440*** | 0.0666 | ← |
| Brazil | 0.2311 | 6.4886** | → |
| Cabo Verde | 0.2661 | 0.1431 | **⇼** |
| Cameroon | 0.8407 | 0.2858 | **⇼** |
| Chile | 2.5497 | 0.0079 | **⇼** |
| China | 1.4534 | 0.1382 | **⇼** |
| Colombia | 0.1221 | 2.4024 | **⇼** |
| Costa Rica | 0.0027 | 0.4041 | **⇼** |
| Cote d'Ivoire | 2.1484 | 0.8328 | **⇼** |
| Cuba | 5.3328** | 1.0655 | ← |
| Dominica | 0.0018 | 0.0092 | **⇼** |
| Dominican Republic | 0.0923 | 1.0385 | **⇼** |
| Ecuador | 0.1633 | 0.0428 | **⇼** |
| Egypt Arab Republic | 0.0888 | 0.4670 | **⇼** |
| El Salvador | 0.0116 | 0.0530 | **⇼** |
| Equatorial Guinea | 0.0059 | 0.0366 | **⇼** |
| Eswatini | 0.2267 | 0.5117 | **⇼** |
| Fiji | 2.9181*** | 3.2710*** | **↔** |
| Gabon | 1.0471 | 0.7044 | **⇼** |
| Ghana | 4.3782** | 0.6589 | ← |
| Grenada | 1.3447 | 0.0225 | **⇼** |
| Guatemala | 1.5714 | 0.0701 | **⇼** |
| Guyana | 0.1954 | 0.0019 | **⇼** |
| Honduras | 0.2508 | 0.4900 | **⇼** |
| India | 3.1687*** | 0.0318 | ← |
| Indonesia | 2.1277 | 0.0491 | **⇼** |
| Iran Islamic Republic | 1.4251 | 0.0693 | **⇼** |
| Iraq | 5.8615** | 2.3926 | ← |
| Jamaica | 2.4453 | 0.0125 | **⇼** |
| Jordan | 0.0424 | 0.0262 | **⇼** |
| Kenya | 1.1342 | 0.2459 | **⇼** |
| Korea Republic | 0.1172 | 0.6928 | **⇼** |
| Lebanon | 0.8060 | 2.8002*** | → |
| Malaysia | 4.1611** | 3.7480 *** | **↔** |
| Marshall Islands | 0.17351 | 1.9510 | **⇼** |
| Mauritius | 0.0067 | 2.6583 | **⇼** |
| Mexico | 0.1343 | 5.235** | → |
| Micronesia Federal States | 0.0801 | 1.0030 | **⇼** |
| Mongolia | 1.0558 | 0.3395 | **⇼** |
| Morocco | 0.0029 | 0.1294 | **⇼** |
| Namibia | 0.7474 | 0.0002 | **⇼** |
| Nicaragua | 0.8521 | 0.5330 | **⇼** |
| Pakistan | 1.5116 | 1.4482 | **⇼** |
| Panama | 0.5201 | 0.1303 | **⇼** |
| Paraguay | 0.1001 | 0.4544 | **⇼** |
| Peru | 0.0123 | 0.1394 | **⇼** |
| Philippines | 0.0013 | 0.0306 | **⇼** |
| Samoa | 0.4539 | 0.1450 | **⇼** |
| Saudi Arabia | 0.0090 | 1.0475 | **⇼** |
| Seychelles | 3.7054 | 0.7748*** | → |
| Singapore | 0.0089 | 0.0965 | **⇼** |
| South Africa | 4.2098** | 0.0312 | ← |
| Sri Lanka | 0.2543 | 3.1648*** | → |
| St. Kitts and Nevis | 0.0007 | 0.0105 | **⇼** |
| St. Lucia | 0.3654 | 2.0068 | **⇼** |
| St. Vincent and the Grenadines | 0.1851 | 0.4654 | **⇼** |
| Syrian Arab Republic | 13.359* | 0.3294 | ← |
| Thailand | 0.0124 | 0.0335 | **⇼** |
| Tonga | 1.281 | 0.2364 | **⇼** |
| Trinidad and Tobago | 0.9841 | 0.1012 | **⇼** |
| Tunisia | 0.0767 | 8.0173* | → |
| Turkey | 0.4635 | 1.6589 | **⇼** |
| United Arab Emirates | 3.2195*** | 0.0745 | ← |
| Uruguay | 2.3304 | 1.1966 | **⇼** |
| Vanuatu | 0.1404 | 1.7228 | **⇼** |
| Vietnam | 1.8668 | 0.2376 | **⇼** |
| Zimbabwe | 0.9206 | 2.3010 | **⇼** |
