# Supplementary material for: The interconnectedness of energy consumption with economic growth: A granger causality analysis
Source: Heliyon. 2024 Aug 28;10(17):e36709. doi: 10.1016/j.heliyon.2024.e36709 (PMC11402754; doi:10.1016/j.heliyon.2024.e36709)

**Appendix L. IRF Graphs for NREC-GDP**

1. Least-developed Countries


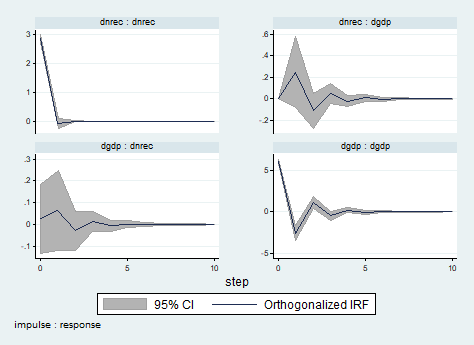


(B) Developed Countries


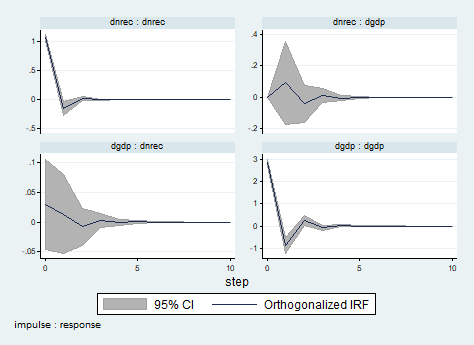


(C) Transitional economies


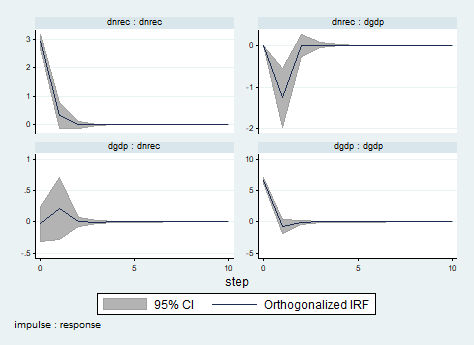


(E) Developing Countries


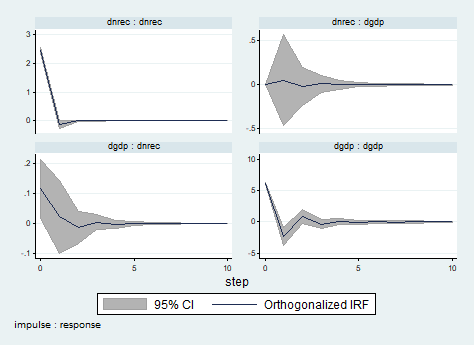


(F) Global view


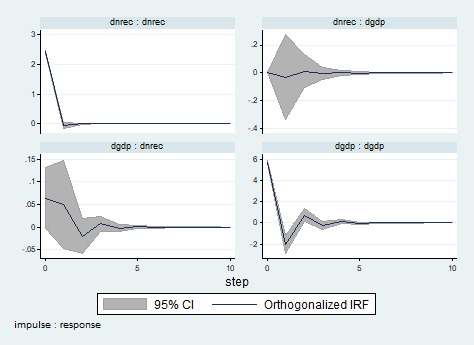

Supplement: Multimedia component 12 [file mmc12.docx]
